# Synthesis of dual-stimuli responsive metal organic framework-coated iridium oxide nanocomposite functionalized with tumor targeting albumin-folate for synergistic photodynamic/photothermal cancer therapy

**DOI:** 10.1080/10717544.2022.2127973

**Published:** 2022-09-26

**Authors:** Xiangtian Deng, Renliang Zhao, Qingcheng Song, Yiran Zhang, Haiyue Zhao, Hongzhi Hu, Zhen Zhang, Weijian Liu, Wei Lin, Guanglin Wang

**Affiliations:** a Trauma Medical Center, Department of Orthopedics Surgery, West China Hospital, Sichuan University, Chengdu, China; b Orthopedics Research Institute, Department of Orthopedics, West China Hospital, Sichuan University, Chengdu, China; c Department of Orthopaedic Surgery, The Third Hospital of Hebei Medical University, Shijazhuang, China; d School of Medicine, Nankai University, Tianjin, China; e Department of Orthopaedics, Union Hospital, Tongji Medical College, Huazhong University of Science and Technology, Wuhan, China; f Department of Gynecology, West China Second Hospital, Sichuan University, Chengdu, China

**Keywords:** Iridium oxide, metal organic framework, photothermal therapy, photodynamic therapy, drug targeting

## Abstract

The synergistic effects of photothermal therapy (PTT) and photodynamic therapy (PDT) has attracted considerable attention in the field of cancer therapy because of its excellent anti-tumor effect. This work provides a novel pH/NIR responsive therapeutic nanoplatform, IrO_2_@ZIF-8/BSA-FA (Ce6), producing a synergistic effect of PTT-PDT in the treatment of osteosarcoma. Iridium dioxide nanoparticles (IrO_2_ NPs) with exceptional catalase-like activity and PTT effects were synthesized by a hydrolysis method and decorated with zeolitic imidazolate framework-8 (ZIF-8) shell layer to promote the physical absorption of Chlorin e6 (Ce6), and further functionalized with bovine serum albumin-folate acid (BSA-FA) for targeting tumor cells. The IrO_2_@ZIF-8/BSA-FA nanocomposite indicated an outstanding photothermal heating conversion efficiency of 62.1% upon laser irradiation. In addition, the Ce6 loading endows nanoplatform with the capability to induce cell apoptosis under 660 nm near-infrared (NIR) laser irradiation through a reactive oxygen species (ROS)-mediated mechanism. It was further testified that IrO_2_@ZIF-8/BSA-FA can function as a catalase and convert the endogenous hydrogen peroxide (H_2_O_2_) into oxygen (O_2_) to improve the local oxygen pressure under the acidic tumor microenvironment (TME), which could subsequently amplified PDT-mediated ROS cell-killing performance via relieving hypoxia microenvironment of tumor. Both in vitro and in vivo experimental results indicated that the nanomaterials were good biocompatibility, and could remarkably achieve tumor-specific and enhanced combination therapy outcomes as compared with the corresponding PTT or PDT monotherapy. Taken together, this work holds great potential to design an intelligent multifunctional therapeutic nanoplatform for cancer therapy.

## Introduction

Cancer, as a leading cause of human deaths worldwide, still remains a great challenge to human health and has become one of the main obstacles to enhancing life expectancy. The current treatment approaches for cancer are mainly limited to traditional chemotherapy, radiation therapy, and surgery, which often cause serious physical harm and undesired side effects on human health. To overcome these drawbacks, many researchers have developed some intelligent strategies with reduced side effects for cancer therapy. Near-infrared (NIR) light-triggered photothermal therapy (PTT) and photodynamic therapy (PDT) has aroused considerable fundamental and translational attention in the field of cancer treatment due to their minimal systemic toxicity, non-invasiveness, and high spatiotemporal selectivity in cancer therapy (Castano et al., 2006; Lovell et al., 2010; X. Li et al., 2018).

PDT, as a typical oxidative therapeutic strategy, is capable of utilizing photo-activated photosensitizing agent (PSA) to transfer the absorption of photo energy to ambient oxygen (O_2_) and subsequently producing high levels of toxic reactive oxygen species (ROS) for killing cancer cells (Dolmans et al., 2003). The anti-tumor therapeutic effect of PDT depends on the concentration of PSA, laser irradiation intensity, and adequate oxygen content. Furthermore, the content of oxygen could directly decrease the therapeutic efficacy of PDT owing to the insufficient O_2_ supply within abnormal tumor vasculature (Tan et al., 2021). More importantly, the rapid consumption of O_2_ during PDT process may further exacerbate the hypoxia microenvironment of tumor, thereby facilitates tumor proliferation, recurrence, metastasis, and invasion, resulting in compromised treatment and poor prognosis (Li et al., 2020). Considering these issues, various therapeutic strategies have been proposed in past decades to modulate tumor hypoxia with the goal of improving the efficacy of PDT (Sun et al., 2020; Hu et al., 2021; Yang et al., 2021). Oxygen-carrying carriers, such as metal-organic framework (MOF) nanocarriers, have been extensively explored for delivering exogenous oxygen to tumor tissue resulting in enhanced PDT efficacy (Gao et al., 2018; Cai et al., 2019). Metal-based nanoenzymes, possessing catalase-like catalytic capability, serves as an intracellular hydrogen peroxide (H_2_O_2_) regulator to enhance PDT efficacy via catalyzing the decomposition of endogenous H_2_O_2_ to generate oxygen and promoting ROS generation (X. L. Liu et al., 2021; Qin et al., 2021; Wang et al., 2021). Despite H_2_O_2_ is overexpressed within tumor microenvironment (TME), the low levels of H_2_O_2_ cannot completely induce tumor cells death, which was because that tumor cells are equipped with antioxidant defense systems. Previous studies have demonstrated that the high levels of H_2_O_2_ within the TME can be utilized as substrate to be converted into molecular oxygen by the nanoenzymes to augment PDT efficacy (Wang et al., 2019; Chen et al., 2021; Wu et al., 2021). Nanoenzymes, as a type of artificial mimic enzymes with both excellent catalytic capacity and superior biocompatibility, is emerging as a promising strategy for cancer therapy (Yu et al., 2020; Cui et al., 2021; Wang et al., 2021). Hence, nanoenzymes can be utilized to exert the ability of self-accelerating continuous generation of oxygen, resulting in an improved efficiency of PDT.

PTT is capable of harness the absorption of the energy from NIR-light and then convert NIR laser into thermal energy through photothermal agents to cause tumor cells death via hyperthermia, which has been rapidly developed and recognized as an attractive treatment for cancers owing to its high selectivity, minimal invasiveness and low side-effects (Xiong et al., 2021; Dai et al., 2022; Qu et al., 2022). The key to realizing photothermic-ablation of tumors depends on the property of photothermal agents which should possess high photothermal stability, promising photo-heat conversion performance, and high biocompatibility. Various types of photothermal agents, including inorganic materials (e.g. noble metal materials (F. Gao et al., 2019; Lv et al., 2021), metal chalcogenide materials (Wu et al., 2020; Curcio et al., 2021), carbon-based nanomaterials (Peng et al., 2017; Qian et al., 2019) and two-dimensional materials (Deng et al., 2021; W. Liu et al., 2021)) and organic materials (e.g. NIR-responsive small molecules (Xu & Pu, 2021) and semiconducting polymer NPs (J. Li et al., 2018; Yin et al., 2020)), have been exploited to perform PTT on tumors in recent years. Unfortunately, it is difficult to realize a comprehensive effect in fighting cancers by mono-PTT. Combinational treatment paradigms have received considerable attention in achieving the therapeutic expectancies of overcoming the shortcomings of monotherapy and realizing well-pleasing outcomes of cancer therapy (Jeong et al., 2021; Wang et al., 2021; Zhang et al., 2021). Therefore, synergistic therapy is an attractive strategy to integrate PTT and PDT into a single nanoplatform to maximized the anti-tumor effects.

Iridium oxide nanoparticles (IrO_2_ NPs) has recently received extensive attention in cancer therapeutic applications owing to its good biocompatibility and remarkable photothermal conversion efficiency (Wu et al., 2020; Li et al., 2021; Yuan et al., 2022). More remarkably, IrO_2_ NPs can function as a catalase and are capable of decomposing endogenous H_2_O_2_ within the TME to generate O_2_ for hypoxia alleviation, thereby amplifying PDT therapy efficacy (Wu et al., 2021; Yuan et al., 2022). Nevertheless, the development of a multifunctional nanoplatform for IrO_2_-based combination of PTT and PDT in cancer therapies is still limited.

Metal-organic framework (MOF), which formed by organic ligands and metal ions or clusters, have gained increasing concern in molecular imaging, drug delivery, and biomedical applications, owing to its advantages of tunable chemical composition, high specific surface area and easy functionalization features (Q. Zheng et al., 2021; Zhou et al., 2021). Of these, zeolitic imidazolate framework-8 (ZIF-8) has been considered as most attractive drug delivery nanoplatform because of its properties of excellent biocompatibility and pH-responsive biodegradability (L. Gao et al., 2019; Shao et al., 2021; Yu et al., 2021). More importantly, zinc ions have a high affinity with photosensitizer Ce6, allowing Ce6 molecular to be incorporated into ZIF-8 by one-pot encapsulation. Considering these unique properties, an intelligent nanoplatform based on biocompatibility and biodegradability was constructed for the combination of PDT and PTT against cancers.

**Figure 1.**
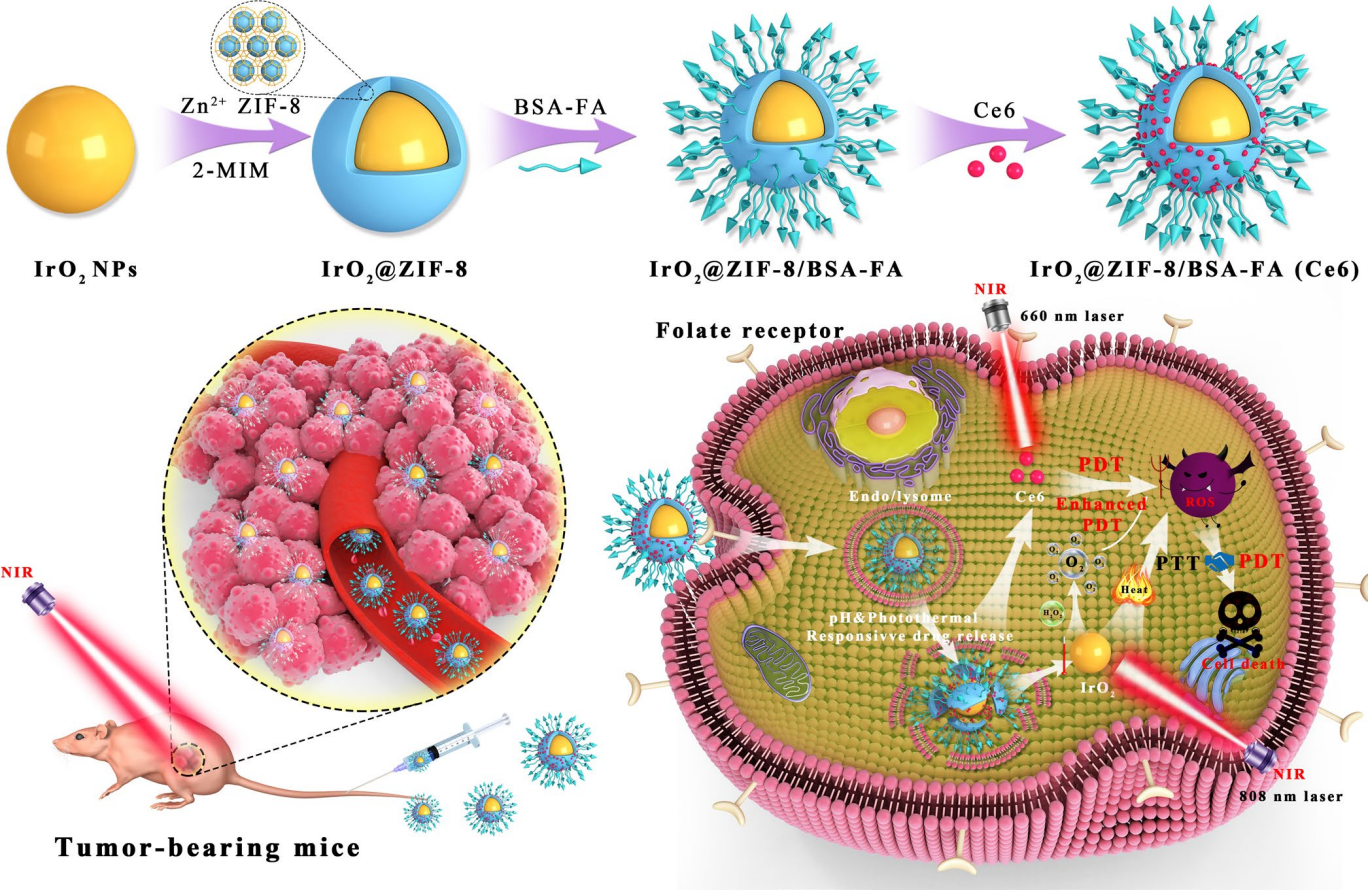
The schematic illustration of the preparation process of IrO_2_@ZIF-8/BSA-FA (Ce6) NPs for the PTT-PDT synergistic cancer therapy in vivo for combination therapy, the mice were first irradiated with an 808 nm laser at 1.0 W cm^−2^ for 10 min, followed by irradiation by a 660 nm laser at 0.5 W cm^−2^ for 10 min.

Consequently, we designed and developed a TME-responsive, bovine serum albumin-folate acid (BSA-FA) functionalized hybrid nanoplatform (IrO_2_@ZIF-8/BSA-FA (Ce6), denoted as IZBFC) in the treatment of cancers, which displays high drug loading capacity and are capable of overcome the shortfalls of Ce6, thereby achieving synergistic therapeutic effects of PTT-PDT for tumor eradication. The prepared process of the IZBFC NPs is illustrated in Figure 1. IrO_2_ NPs were synthesized through facile hydrolysis of IrCl_3_, and polyvinylpyrrolidone (PVP) was used as a template for the formation of IrO_2_-PVP. Subsequently, ZIF-8 shell layer was encapsulated on the surface of IrO_2_ to facilitate the physical absorption of Ce6, followed by the conjugating of BSA-FA on the surface of IrO_2_@ZIF-8 as an active targeting agent to form IrO_2_@ZIF-8/BSA-FA (Ce6). Moreover, the nanoplatform properties results indicated that low pH stimulus and photothermal performance facilitated degradation of outer shell layer, providing a basis for releasing the loaded drugs in a controlled manner. At the same time, NIR-induced local hyperthermia is not only contributed to promoting Ce6 release, but also amplifying ROS-induced cell-killing ability, which exhibited comprehensive anti-tumor effects. Apart from this, IrO_2_ catalyzes endogenous H_2_O_2_ to achieve in-situ oxygen supply and enhances Ce6-mediated PDT. More importantly, systematic in vitro and in vivo evaluations have showed that IZBFC NPs exerted good biocompatibility and were capable of actively accumulate into tumors via FA-mediated targeting, thereby achieving the synergistic anti-tumors effects of PTT and PDT. The present study offers an intelligent design of IrO_2_@ZIF-8/BSA-FA nanocomposite for combined PTT and PDT, which synergistically enhance the efficacy of PTT-PDT for tumor eradication and possess important potential for cancer treatment.

## Experimental section

### Synthesis of PVP-modified IrO_2_ NPs

The preparation of PVP-modified IrO_2_ was prepared as described in a previously literature (Wu et al., 2021). Firstly, 0.5 g IrCl_3_ and 1.0 g PVP were added into distilled water (150 ml), followed by magnetic stirring (400 rpm). After that, NaOH solution (2.0 M, pH = 12) were poured into the mixture. After mixing for 12 h (at 80 °C) under continuous stirring, IrO_2_-PVP NPs were collected by centrifugation at 16,000 rpm for 5 min, washed several times with ethanol and distilled water, and then stored at 4 °C for later use.

### Synthesis of IrO_2_@ZIF-8 NPs (IZ) NPs

IZ NPs was prepared according to the following route. Firstly, the as-synthesized IrO_2_-PVP dispersion (2 ml, 4.0 mg/mL) was mixed with 1485 μL of Zn(NO_3_)_2_·6H_2_O solution (1 μg/mL) under string, following by addition of aqueous 2-MIM solution (1 mg/mL, 2870 μL). After being reaction for 20 min, IZ NPs was obtained by centrifugation at 16,000 rpm for 5 min and washed with several times with deionized water.

### Synthesis of IrO_2_@ZIF-8/BSA-FA (IZBF) NPs

IZBF NPs was synthesized by facile functionalization of IZ NPs with BSA-FA. The preparation of BSA-FA was based on a previous study (Ma et al., 2017). According to the UV-vis spectra analysis (Figure S1), FA represented obvious peaks at 291 nm and 363 nm, and the absorption of FA-BSA at 363 nm confirmed the successful synthesis of BSA-FA. The obtained IZ NPs were dispersed in BSA-FA alkali aqueous solution (5 ml) and stirred for 40 min. Then, the resulting IZBF NPs were isolated by centrifugation, washed with deionized water at least three times, and then the product was free-dried and stored at 4 °C for later use.

### Synthesis of IrO_2_@ZIF-8/BSA-FA (Ce6) (IZBFC) NPs

The Ce6 was absorbed onto the surface of IZBF NPs by simple mixing. Briefly, 5 mL of Ce6 (2 mg/mL in DMSO) was first mixed with the above synthesized products IZBF NPs (5 mg) by ultrasonication, followed by stirring for 24 h at room temperature. Finally, IZBFC NPs were obtained by centrifugation (15,000 rpm, 5 min). To assess the Ce6 loading content (LC) and encapsulation efficiency (EE), the standard curve of Ce6 was plotted with a UV-vis spectrum (Shimadzu UV3600). The Ce6 LC and EE of IZBFC NPs were calculated according to the equation (Huang et al., 2021): LC_Ce6_ = (Weight of Ce6 in NPs)/(Weight of Ce6 in NPs + Weight of NPs) × 100%; EE _Ce6_ = (Weight of Ce6 in NPs)/(Weight of initial Ce6) × 100%.

### pH/NIR dual stimuli-responsive drug release behavior

The Ce6 release behaviors from IZBFC were evaluated using the dialysis method in four different medial, including: (1) pH 7.4, (2) pH 7.4 + 808 laser irradiation, (3) pH 5.0, and (4) pH 5.0 + 808 laser irradiation. The IZBFC (2 mg) was diluted to 1 mg/mL, 1 ml of which dialyzed against 30 mL of PBS at the above different conditions for 10 min at 37 °C in a shaking bath. At predetermined time intervals, the amounts of released Ce6 from the IZBFC NP in the supernatant solutions were obtained with a UV-Vis spectroscopy.

### Measurement of the dissolved oxygen content

To investigate the oxygen generation under simulated TME conditions, the catalase-like catalytic efficiency of IZBF NPs was evaluated by mixing IZBF NPs with PBS buffer (pH= 6.0) containing 1 mM H_2_O_2._ At the designed time intervals, the generation of oxygen in aqueous solution was evaluated using the dissolved oxygen meter.Meanwhile, the IZBF NPs in the absence of H_2_O_2_ was set as a control.

### Light conversion capacity of IZBF

To investigate the photothermal property, 200 μL of IZBF suspension in water was prepared in various Ir concentrations (0, 0.5, 1.0, 3.0 and 6.0 mM) were added into a centrifugation tube and irradiated using an 808 nm laser with a power density of 1.0 W/cm^2^ over a period of 600 s. To further evaluate the effect of power density-dependent thermal characteristics on temperature variations, the IZBF NPs dispersion (Ir: 3 mM) was irradiated with a different power density (0.4, 0.8, 1.2, 1.6 and 2.0 W/cm^2^) for 600 s. The real-time temperature changes at the predetermined time points ranging from 0 to 10 mins and the corresponding temperature of solution was immediately captured by a thermal imaging camera (Testo 865, Testo, Schwarzwald, Germany). Also, the temperature variation of IZBF was evaluated under periodic laser exposure (808 nm, 1 W/cm^2^) for five on/off cycles. In brief, laser irradiation against centrifugation tube was performed for 10 min, followed by spontaneously cooling down to ambient temperature of surroundings before the next cycle. Besides, the photothermal conversion efficiency (η) of IZBF NPs was determined according to a previous reported method (Huang et al., 2021).

### In vitro biocompatibility assay

The biocompatibility of nanocarrier, including cytocompatibility and hemocompatibility, is a prerequisite for biomedical applications of nanomaterials (Singh et al., 2021). Accordingly, biocompatibility of IZ and IZBF NPs was investigated by calculating the cell viability of BMSC cells (Bone marrow stromal cells). Briefly, the cells were seeded in 96-well plate with a density of 5000 cells/well and cultured at 37° C for 24 h_._ Subsequently, the adherent cells were exposed to IZ and IZBF at a wide range of concentrations (0–800 μg/mL) for another 24 h and the untreated cells were set as the negative control. After that, 10 μL of cell counting kit-8 (CCK-8) detection solution was further added into each tested well and then incubated for another 4 h. Finally, the plate was evaluated using a microplate reader at the wavelength of 450 nm to calculate the cell viability.

The hemocompatibility of IZBF NPs was evaluated by the standard in vitro hemolytic assay. In brief, free blood samples (2 mL) was obtained from Balb/c mice and centrifuged at 3500 rpm for 5 min at 4 °C to obtain the mice erythrocyte. Afterwards, the precipitated erythrocyte was washed with PBS three times and re-suspended in PBS. To evaluate the hemolytic effect, the above erythrocyte dispersions (200 μL) was mixed with a series of concentrations (25, 50, 100, 200, 400 and 800 μg/mL) of IZBF and cultured at 37 °C. At the same time, erythrocyte dispersions (0.2 mL) were incubated with 1 mL of PBS solution (negative control) and 1 mL DI water (positive control) respectively. After incubating for 4 h, the above mixed solution was centrifugated at 3500 rpm for 5 min, and the supernatants were extracted. After that, the absorbance of the supernatant at 541 nm was recorded to calculate the hemolysis according to the study.

### Cellular uptake

MNNG/HOS cells (1 × 10^5^ cells per well) were seeded in 6-well plates, treated with IrO_2_@ZIF-8/Ce6 and IZBFC for 4 h. At the same time, competitive inhibition experiment was also conducted. Briefly, cells were pretreated with free FA (2 mg/mL), followed by incubation with IZBFC NPs for 4 h. The cells were then rinsed with PBS for three times, and Hoechst 33,342 (10 μg/mL) was added and unbated with cells for 10 min. Cellular uptake can be explored by intracellular Ce6 fluorescence. Images were obtained using a fluorescence microscopy.

### In vitro synergistic PTT/PDT

To evaluate the synergistic effects of PTT and PTT of IZBFC NPs in vitro, MNNG/HOS cells (5000 cells per well) were plated into 96-well plates and incubated for 24 h with 5% CO_2_ at 37° C allowing cell attachment. Afterwards, PBS and IZBFC were added to the plates, and the cells were incubated for 24 h. For mono-laser group without the addition of NPs, the cells were first irradiated with 808 nm laser (1.0 W cm^−2^) for 10 min, followed by irradiated with 660 nm laser (0.5 W cm^−2^) for 10 min. For mono-PTT group, the cells were irradiated with 808 nm laser (1.0 W cm^−2^, 10 min). For mono-PDT group, the cells were irradiated with 660 nm laser (0.5 W cm^−2^, 10 min). For synergistic PTT + PDT group, the cells were first irradiated with 808 nm laser (1.0 W cm^−2^, 10 min), and then irradiated with 660 nm laser (0.5 W cm^−2^, 10 min). Cells without laser exposure were set as the control group. Finally, the cell viabilities were evaluated by CCK-8 assay. The synergistic effect of PTT/PDT was evaluated by combination index (CI) analysis (Xu et al., 2018), for which the CI value of PTT/PDT can be calculated by the following formula: CI = D_1_/D_m1_+ D_1_/D_m2_, where D_1_ is the half-maximal inhibitory concentration (IC_50_, 50% reduction in cell viability) when PTT and PDT produced a specified effect together, D_m1_ and D_m2_ are the doses with the same effect for a single therapy.

### Live/dead cell staining assay

Live/deal cell staining assay was performed to investigate the cell growth inhibition of different treatments. In brief, 6-well plates full of MNNG/HOS cells (1 × 10^5^ cells per well) were treated with the corresponding treatments, and all groups of cells were stained with both calcein AM (calcein acetoxymethyl ester) and PI (propidium iodide) to visualize the therapeutic efficacy.

### Intracellular reactive oxygen species (ROS) generation analysis

2′, 7′-dichlorodihydrofluorescein diacetate (DCFH-DA) is a fluorescent marker for intracellular ROS, which can be oxidized by ROS and emits bright green fluorescence. In brief, MNNG/HOS cells (1 × 10^5^ cells per well) were first inoculated in a 6-well plate and incubated at 37° C in 5% CO_2_ overnight. After 4 h incubation, the cells were divided into five groups according to the different therapeutic formulations: (1) PBS; (2) 808 nm + 660 nm laser; (3) IZBFC + 808 nm laser (1.0 W cm^−2^, 10 min); (4) IZBFC + 660 nm laser (0.5 W cm^−2^, 10 min); and (5) IZBFC + 808 nm + 660 nm laser, respectively. After incubation for another 4 h, the cells were rinsed with PBS three times, followed by the addition of DCFH-DA solution (10 μM) and incubated for another 30 min. After that, the cells were rinsed with PBS three times and then observed under a fluorescence microscopy.

### Cell apoptosis

Cell apoptosis assay against MNNG/HOS cells was also evaluated using Annexin V-fluorescein isothiocyanate (FITC)/propidium iodide (PI). Briefly, MNNG-HOS cells (1 × 10^5^) were seeded on a 6-well plate for 24 h, followed by rinsed with PBS three times and the cells were treated with IZBFC for 4 h, and then irradiated with 808 nm and/or 660 nm laser. After 12 h of incubation, the cells were treated with Annexin V-FTIC/PI reagent according to the manufacturer’s protocol, and then analyzed by flow cytometry.

### Animals and tumor model

Animal experiments were executed in accordance with the Guide for the Care and Use of Laboratory Animals and were approved by Institutional Animal Care and Use Committee of Sichuan University. MNNG/HOS tumor bearing Balb/c nude mice were elected as the animal tumor models in this study. In brief, 200 μL of PBS containing MNNG/HOS cells (1 × 10^7^) was subcutaneously injected into right flank of Balb/c mice. Subsequently, the animal tumor model was continuously fed till a tumor volume of about 100 mm^3^.

### In vivo biodistribution and blood circulation

When the tumor size attained to approximately 100 mm^3^, the tumor-bearing mice (*n* = 3) were intravenously administrated with IZBFC NPs (200 μL, 20 mg/kg). About 20 μL of blood was extracted from the treated mice at predetermined time intervals (1, 2, 4, 8, 12, and 24 h), weighted and then diluted in digesting aqua regia (HNO_3_: HCl = 1:3). The amounts of Ir in the blood were analyzed through ICP-AES. On the hand, to obtain the in vivo biodistribution study, the tumor-bearing mice (*n* = 3) were intravenously injected with IZBFC NPs (200 μL, 20 mg/kg). Afterwards, the mice were sacrificed and the content of Ir elements in tumors and major organs (heart, liver, spleen, lung, and kidney) were measured at different time points.

### In vivo anti-tumor activity

When the tumor volume of 100 mm^3^ attained, the mice were randomly allocated into 5 groups (5 mice for each group) randomly and treated with: (1) PBS; (2) laser; (3) IZBFC + 808 nm laser (1.0 W cm^−2^, 10 min); (4) IZBFC + 660 nm laser (0.5 W cm^−2^, 10 min); and (5) IZBFC + 808 nm + 660 nm laser. For combination therapy, the mice were first irradiated with an 808 nm laser at 1.0 W cm^−2^ for 10 min, followed by irradiation by a 660 nm laser at 0.5 W cm^−2^ for 10 min. Also, the local temperature of tumor was monitored using thermal infrared imaging camera. The anti-tumor effects were further investigated by recording the tumor volume and body weight changes. Tumor volumes were determined every two days by the following equation: Tumor Volume (V) = [(length) × (width)^2^]/2.

### In vivo biocompatibility assay

To determine the biocompatibility of IZBFC NPs, Balb/c mice were administrated with intravenous injection of IZBFC NPs (200 μL, 20 mg/kg) and the mice treated with PBS were set as the negative control. After 14 days corresponding treatment, all mice were sacrificed, and the organs and tumors were applied for H&E staining and visualized microscopically for histological analysis. The tumor slices were also analyzed by TdT-mediated dUTP nick-end labeling (TUNEL) staining for cell death and Ki-67 immunohistochemistry for proliferation according to the manufacture’s protocol. In addition, their blood was collected for blood biochemistry test.

### Statistical analysis

SPSS software was used for the statistical data analysis. Data were expressed as mean ± standard deviation (SD). One-way analysis of variance (ANOVA) was performed to determine statistical significance of the data. *p* < 0.05 was considered statistically significant.

## Results and discussion

### Preparation and characterization of the IZBF NPs

The preparation process of the IZBF NPs and its synergistic anti-tumor mechanism was shown in Figure 1. In brief, PVP-modified IrO_2_ NPs (IrO_2_-PVP) were synthesized via facile hydrolysis of IrCl_3_, and a purple-blue IrO_2_ aqueous solution was obtained (Figure S2). The ZIF-8 shell layer was in-situ grew on the surface of IrO_2_ by mixing 2-MIM with the PVP-functionalized IrO_2_ NPs in solution owing to the hydrogen bond between 2-MIM and PVP; thus, ZIF-8 was successfully capped around IrO_2_ NPs to form the IrO_2_@ZIF-8 (IZ) NPs with the introduction of Zn^2+^ (Figure S3). Subsequently, BSA-FA conjugation was fabricated through amide interaction as the active tumor-targeting ligand and then coated on the surface of the IZ NPs to form IZBF NPs by interaction between IZ NPs and protein.

**Figure 2.**
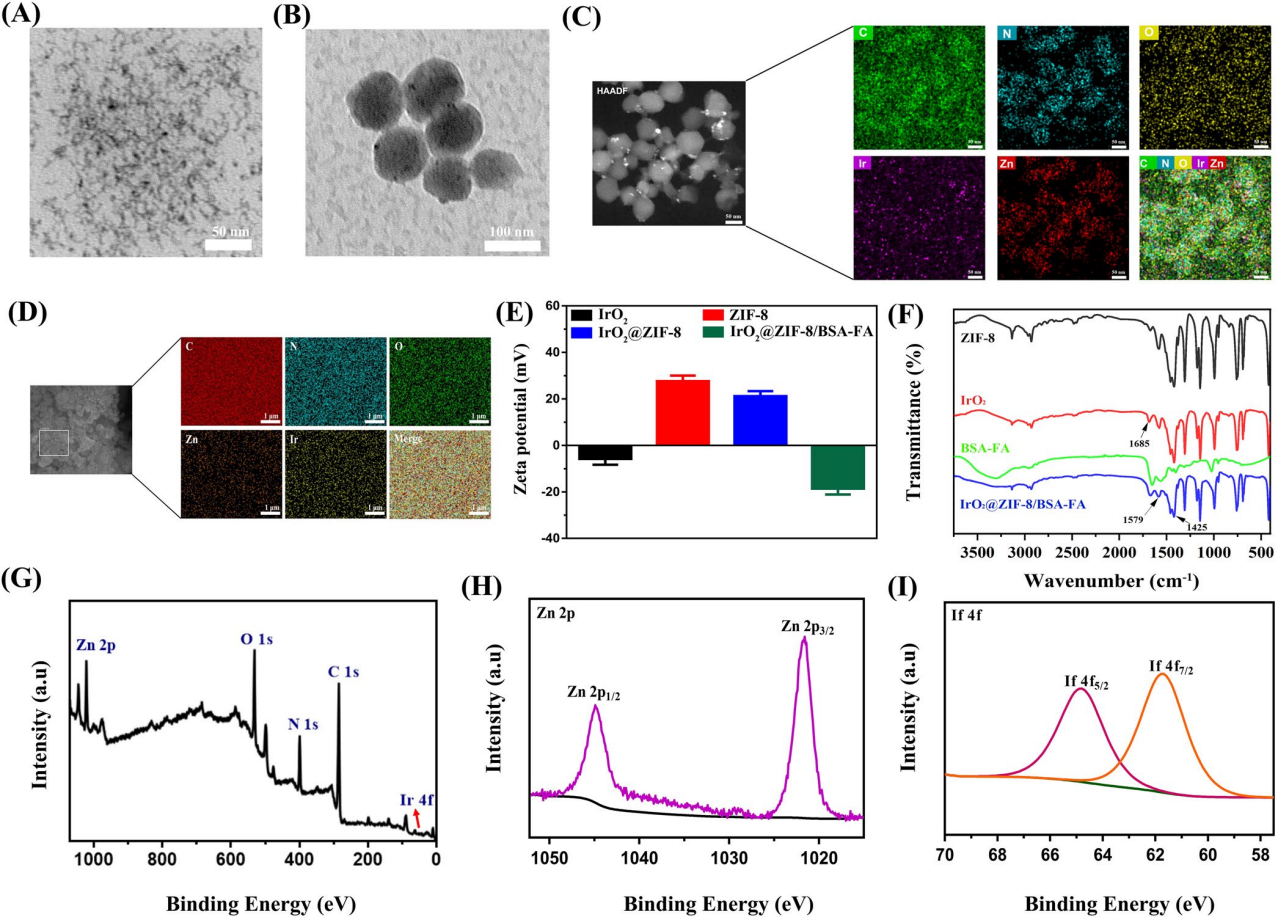
The characterization analysis of the as-prepared formulations. TEM images of (A) IrO_2_-PVP NPs and (B) IrO_2_@ZIF-8/BSA-FA NPs. (C), (D) TEM mapping and SEM mapping of the IrO_2_@ZIF-8/BSA-FA NPs. (E) Zeta potentials of IrO_2_, ZIF-8, IrO_2_@ZIF-8 and IrO_2_@ZIF-8/BSA-FA NPs, and (F) FTIR spectra of ZIF-8, IrO_2_, BSA-FA, IrO_2_@ZIF-8/BSA-FA NPs. XPS spectra of (G) IrO_2_@ZIF-8/BSA-FA, (H) Zn 2p and (I) Ir 4f.

**Figure 3.**
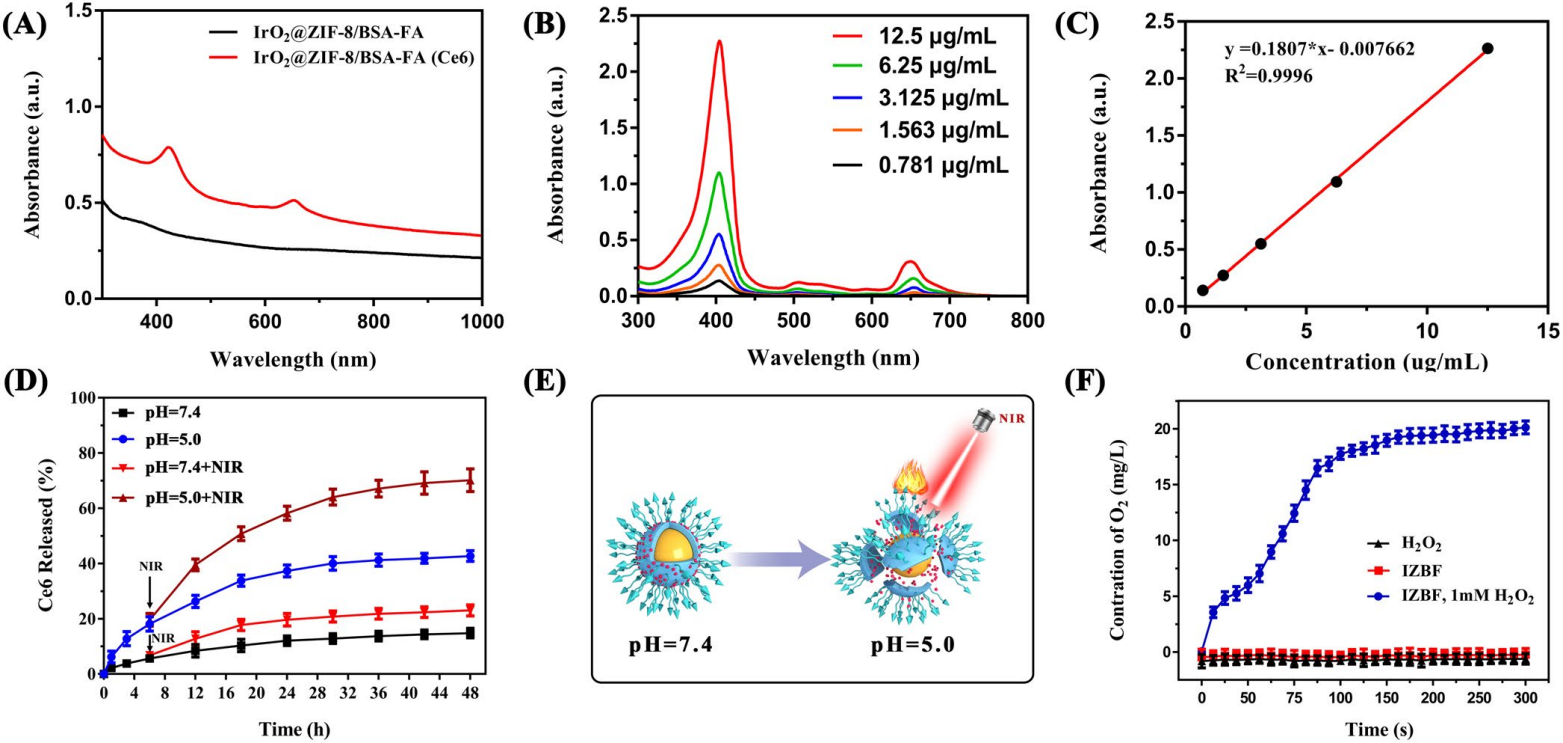
(A) Light absorption of IrO_2_@ZIF-8/BSA-FA and IrO_2_@ZIF-8/BSA-FA (Ce6). (B) The UV-vis absorption spectra of Ce6 with different concentrations. (C) Standard curve of Ce6. (D) Cumulative release of Ce6 from IrO_2_@ZIF-8/BSA-FA (Ce6) nanocomposites in PBS with different pH values in the presence or absence of NIR irradiation (808 nm, 1.0 W cm^−2^, 5 min). (E) Schematic illustration of the pH and NIR-laser triggered release behavior of IZBF NPs. (F) O_2_ generation in H_2_O_2_ solutions (1 mM) after incubation with IZBF NPs.

The morphology of the product was characterized by TEM and SEM. TEM images demonstrated that the synthesized IrO_2_ NPs exhibited a diameter of approximately 13.2 nm (Figure 2(A)), and IZBF NPs showed well-defined spherical-like structure with average size of 74.6 nm (Figure 2(B)). The TEM and SEM elemental mapping also confirmed that the IrO_2_ NPs were encapsulated into ZIF-8 and successfully modified by BSA-FA, demonstrated the coexistence of C, N, O, Ir, and Zn elements (Figure 2(C) and (D)). Additionally, the zeta potentials of IrO_2_, ZIF-8, IZ NPs, and IZBF NPs were −5.7 mV, 27.6 mV, 21.2 mV, and −18.5 mV respectively (Figure 2(E)). The corresponding changes in the zeta potential of the modification step also confirm a successive conjugation of BSA-FA. DLS results revealed that the average hydrodynamics particles size of IZBF NPs were 100.2 nm (Figure S3), which was larger than the hydrodynamic diameter measured by TEM. The difference in size of IZBF NPs between TEM and DLS measurement was probably attributed to the IZBF NPs swelled in water. Moreover, no significant DLS changes and zeta potential were observed for 7 days of dialysis (Figure S5), demonstrating remarkable physiological stability of IZBF.

The chemical structure of IZBF NPs was investigated via FTIR analysis (Figure 2(F)). The characteristic absorption peak at 1685 cm^−1^ indicates the C = O stretching bond vibration of PVP, demonstrating the IrO_2_ NPs successfully embedded in the framework of ZIF-8. In comparison with the IrO_2_ spectrum, obvious bands appeared at 1425 and 1579 cm^−1^ in the IZBF NPs spectrum were indexed to the stretching vibration peak of C-N and amide II absorption of the N-H bond. These findings clearly testified that the BSA-FA conjugation was decorated onto the IZ surface. In addition, the XPS spectra of IZBF NPs showed all the characteristic peaks of C 1 s, N 1 s, O 1 s, Zn 2p, and Ir 4f, further demonstrating the elemental composition of IZBF NPs (Figure 2(G)). Of note, the peaks of Zn 2p (1045.2 and 1022.2 eV) and Ir 4f (65.1 and 61.8 eV) could be attributed to Zn^2+^ and Ir^4+^ (Figure 2(H) and (I)). Collectively, these results demonstrated that the IrO_2_ NPs were successfully coated by ZIF-8 and BSA-FA.

### Drug loading and controlled releasing properties

The poor water solubility and inferior targeting property seriously hampered the application of Ce6. Accordingly, TME-responsive drug delivery carrier (IZBF NPs) was constructed and regarded as vector for loading Ce6. From the results of UV-vis spectra (Figure 3(A)), the as-prepared IZBFC NPs exhibited a characteristic absorption peak at 404 nm and 665 nm when compared to the spectrum of the IZBF NPs, proving the successful encapsulation of Ce6. According to the absorption spectra of different concentrations of Ce6 at 404 nm (Figure 3(B)), the standard curve of Ce6 was plotted (Figure 3(C)). Afterwards, the LC of Ce6 was thus calculated to be about 53.5 ± 3.2% and the corresponding EE was 32.1 ± 3.3%.

The ZIF-8 NPs have been used as desirable nano-vehicles with respect to their intrinsic advantages of pH-responsive degradation capability. As shown in Figure 3(D), only 14.8% of Ce6 was released in pH 7.4, while the accumulative released of Ce6 increased up to 42.7% in pH 5.0 under the same condition, indicating that the pH-dependent release behavior of Ce6, which was ascribed to the degradation of ZIF-8 shell layer in acidic-TME. The negligible release of Ce6 at neutral physiological conditions demonstrated that IZBFC are safe and has great potential for drug delivery. In addition, the NIR-stimulated release behavior of Ce6 was further evaluated at pH 7.4 and pH 5.0. Upon the NIR irradiation, the release rates of Ce6 were slowly increased at pH 7.4, while the Ce6 is quickly released and the cumulative release drastically increased to 70% at pH 5.0. The NIR-triggered Ce6 release from IZBFC NPs might be ascribed to the heat energy producing from IrO_2_ NPs under NIR laser irradiation, which could facilitate degradation of ZIF-8 shell layer and then lead to Ce6 molecules released from the nanocomposite (Figure 3(E)). Therefore, the abovementioned results indicated that the acidic-TEM and NIR-laser irradiation could serve as an effective nanoplatform to control the release of Ce6, thereby avoiding the premature leakage of Ce6 and minimizing toxic side effects to normal tissues

### Catalytic activity of IZBF NPs

The IrO_2_ has been reported to have catalase-like activity, which will be beneficial for enhancing the efficiency of oxygen-consuming PDT by in-situ triggering the catalytic decomposition of H_2_O_2_ to produce oxygen (Yuan et al., 2022). To prove the ability of O_2_ generation, we investigated the dissolved O_2_ content after incubating the IrO_2_@ZIF-8/BSA-FA NPs with H_2_O_2_. As shown in Figure 3(F), the oxygen concentration of the IZBF and H_2_O_2_ as the control group demonstrated no significant changes and remained consistently close to zero. In contrast, we observed that significant amounts of O_2_ were produced by IZBF in PBS solutions containing 1 mM of H_2_O_2_, demonstrating excellent catalase-like activity, which provided a basis for the subsequent PDT to provide a sufficient oxygen source.

### Photothermal capacity of IZBF NPs

**Figure 4.**
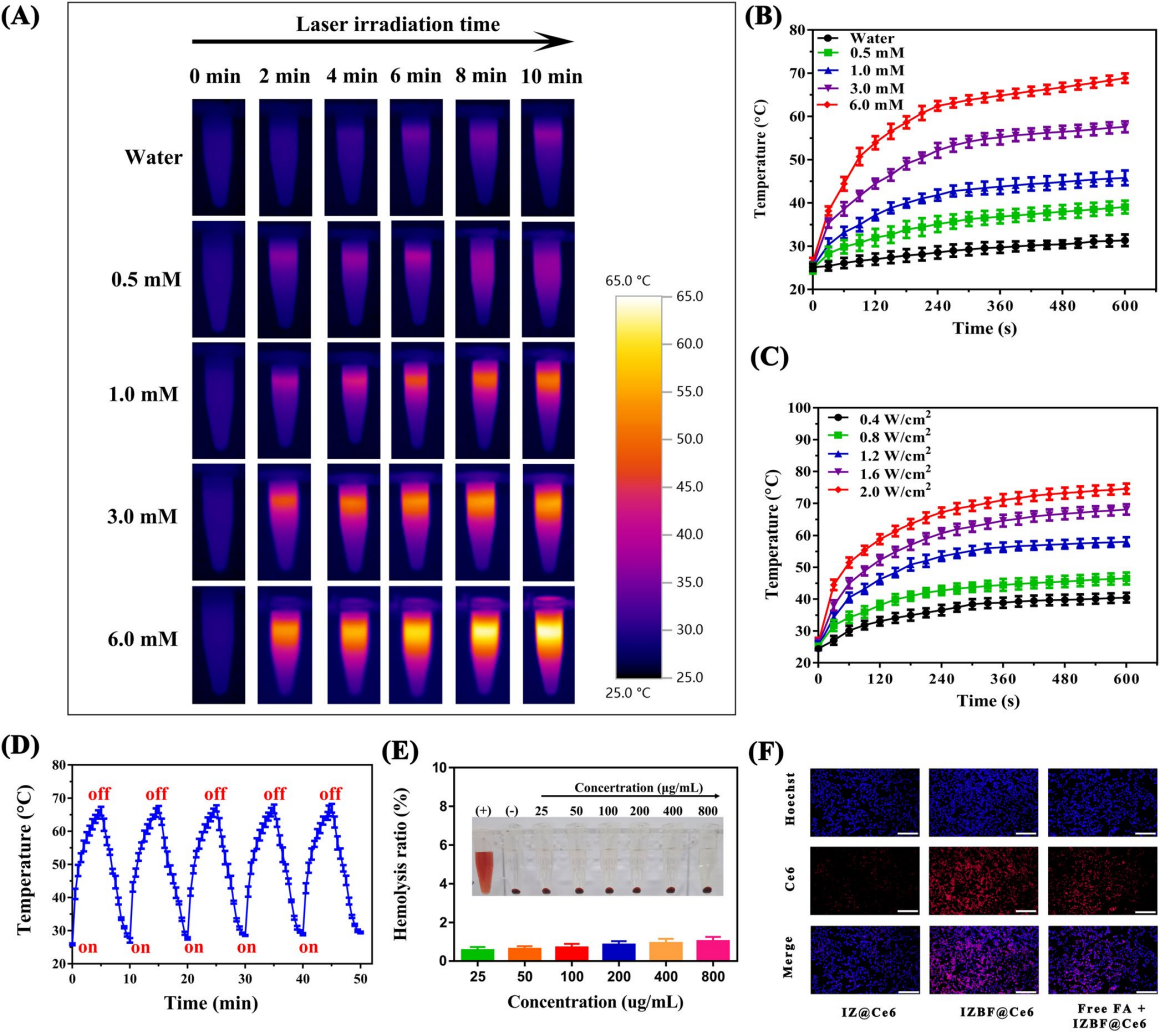
(A), (B) The infrared thermal images and time-dependent temperature changes of IZBF NPs with different concentrations under NIR laser irradiation (808 nm, 1.0 W cm^−2^). (C) The photothermal effect of IZBF as a function of NIR laser irradiation time (Ir: 3 mM) at varied power densities. (D) The photothermal stability of IZBF over five cycles NIR laser irradiation. (E) Hemolysis assay of different concentrations of IZBF NPs. The inset images of tubes containing red blood cells solution show the direct observation of hemolysis. (F) Fluorescence microscopy images of MNNG/HOS cells after incubation with different formulations (Scale bar: 100 μm).

To investigate the photothermic capacity of our designed IZBF NPs, the temperature variation was dynamically recorded by using a digital thermometer at intervals of 30 s. Simultaneously, photothermal imaging were captured by using a thermal imaging camera. Evidently, the temperature of IZBF NPs dispersion showed a distinct concentration- and time-dependent feature (Figure 4(A) and (B)). However, there were only slight temperature changes after PBS irradiated under the same environment. Moreover, it could be seen that the IZBF NPs exhibited an obvious laser power intensity-dependent feature (Figure 4(C)). The remarkable capacity of photothermal effects may be ascribed to the excellent photothermal agents (IrO_2_), converting luminous energy into local hyperthermia. Moreover, the photostability of IZBF NPs was assessed by periodic NIR laser exposure (1.0 W cm^−2^, 10 min) for consecutive five cycles. Notably, the IZBF NPs exhibited superior photothermal stability after laser exposure under five cycles of heating and cooling process (Figure 4(D)). According to the Lambert-Beer law, the extinction coefficient (ε) of NPs was calculated to be as high as 9.17 L g ^− 1 ^cm^−1^ at 808 nm, demonstrating the strong NIR absorption capacity of samples (Figure S6), which would significantly favor NIR laser-activated hyperthermia generation.

Furthermore, the photothermal conversion efficiency (η) of the IZBF NPs was further evaluated based on the previous study (Huang et al., 2021). Therefore, the photothermal conversion efficiency of the IZBF NPs was determined to be approximately 62.1% (Figure S7A and B). In addition, the corresponding temperature changes of IZBFC NPs during the heating and cooling process was shown in Figure S7C. Collectively, our designed IZBF NPs showed excellent photothermal conversion performance and superb thermostability, indicating great application potential as outstanding photothermal agents for cancer treatment.

### Biocompatibility of nanoparticles

The low toxicity or nontoxicity of nanoparticles is an essential prerequisite for biomedical applications of nanocarriers. The cytocompatibility of IZ and IZBF NPs was assessed on BMSCs by using CCK-8 assay. Distinctly, no obvious cytotoxicity to BMSCs was observed after incubation with IZ and IZBF NPs at any tested concentrations (Figure S8), demonstrating the good biocompatibility and low cytotoxicity of the nanocarriers within experimental dosages. Moreover, the hemocompatibility of the nanoparticles was also evaluated by hemolysis assay. As illustrated in Figure 4(E), no noticeable hemolysis rate of erythrocyte in the PBS and IZBF groups was observed even if the concentration was up to 800 μg/mL. Comparatively, pure water (positive control) can cause severe erythrocyte hemolysis. Therefore, the as-synthesized IZBF NPs presented good blood compatibility, which has an enormous potential application in cancer therapy, thereby reducing the side effects of systematic administration.

### Cellular uptake

An essential requirement of nanomaterial-based drug delivery system is highly effective targeted delivery the therapeutic agents into tumor regions. As shown in Figure 4(F), the IZBF@Ce6 nanocomposites showed stronger red fluorescence intensity than that IZ@Ce6 group. The difference in fluorescence intensity between IZ@Ce6 group and IZBF@Ce6 group may can be attributed to the targeting ability of the FA molecule, which can achieve active targeting to FA receptor overexpressed OS cells. In addition, it could be seen that the uptake of IZBF@Ce6 was evidently decreased when pretreated with free FA molecule. These findings can be ascribed to the fact that our developed FA-modified drug delivery system could be efficiently uptake by tumor cells, thus facilitating its accumulation into tumor sites.

### In vitro PTT-PDT synergistic effect of IZBFC NPs

**Figure 5.**
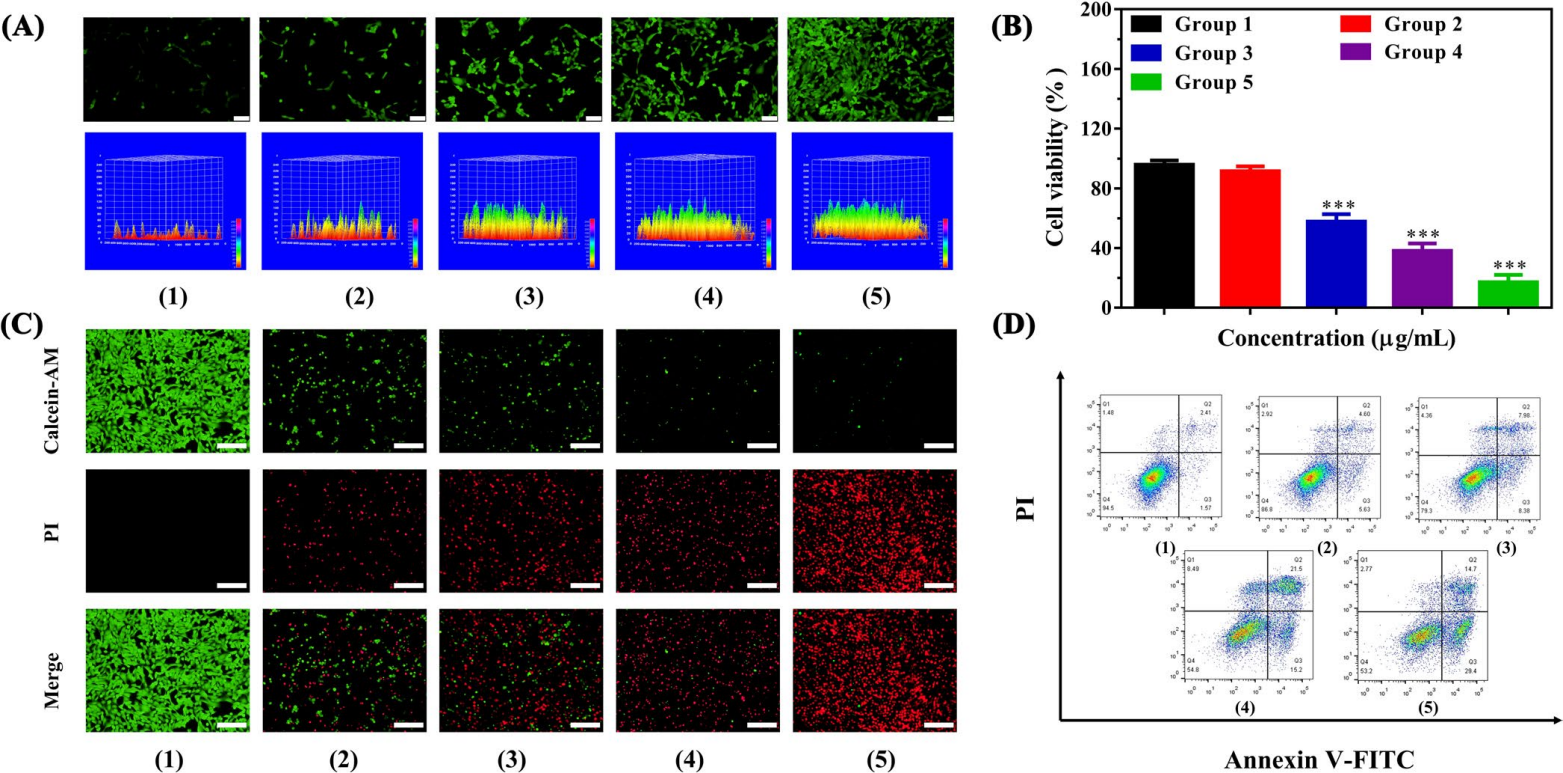
(A) Fluorescence microscopy and corresponding surface plot images of ROS production under the presence of DCFH-DA in MNNG/HOS cells after corresponding treatment for 24 h (Scale bar: 100 μm). (B) Cell viability of MNNG/HOS cells after different treatments. (C) Calcein-AM/PI staining images of MNNG/HOS cells after different treatments (Scale bar: 200 μm). (D) Flow cytometry apoptosis experiment for annexin V-FITC/PI co-stained MNNG/HOS cells after different treatments (Groups included (1) control, (2) 808 + 606 nm laser irradiation, (3) IZBFC + 808 nm laser irradiation, (4) IZBFC + 660 nm laser irradiation, and (5) IZBFC + 808 nm + 660 laser irradiation, respectively).

The synergistic PTT-PDT therapeutic effects of IZBFC NPs were evaluated. DCFH-DA fluorescent probe was selected for detecting the intracellular ROS-generated ability of IZBFC NPs. As presented in Figure 5(A), the IZBFC + 808 + 660 laser-treated MNNG/HOS cells exhibited the strongest fluorescence intensity, which can be explained by the synergistic effects of PTT-PDT. Besides, in vitro therapeutic effects were quantitatively evaluated in MNNG/HOS cells using the CCK-8 assay. Firstly, MNNG/HOS cells were treated with different therapeutic formulations, and then H_2_O_2_ (0.2 mM) was added to mimic the TME. As shown in Figure 5(B), it could be seen that the viability of the MNNG/HOS cells in group 3 decreased to 57.7%, owing to the outstanding photothermal conversion performance of IZBFC NPs. In comparing with control group, the viability of the MNNG/HOS cells in group 4 reduced to 38.2%. This may be ascribed to that the decomposition of H_2_O_2_ into endogenous O_2_ mediated by the catalytic ability of IZBFC NPs. Notably and importantly, it was found that over 80% of the MNNG/HOS cells were killed after being irradiated with 808 nm and 660 nm laser light successively, which can be ascribed to the combined effects of PTT and PDT. The combination index (CI) values of PTT and PDT was calculated to be 0.67, indicating a synergistic effect. Furthermore, similar results were also observed in live/dead staining experiment (Figure 5(C)). Notably and importantly, the red fluorescence in the IZBFC + 808 + 660 nm laser exposure was evidently stronger than that of other groups, validating the promising anti-tumor effects of the synergistic PTT/PDT. Afterwards, quantitative identification of apoptosis caused by IZBFC NPs was further investigated by annexin V-FITC/PI staining (Figure 5(D)). Similarly, it could be seen that the combination group could induce a prominently higher levels of apoptotic cells when compared to other groups (Figure S9), demonstrating that the multifunction nanoplatform can enhance the anti-tumor therapeutic efficacy of PTT and PDT.

### In vivo photothermal effect

**Figure 6.**
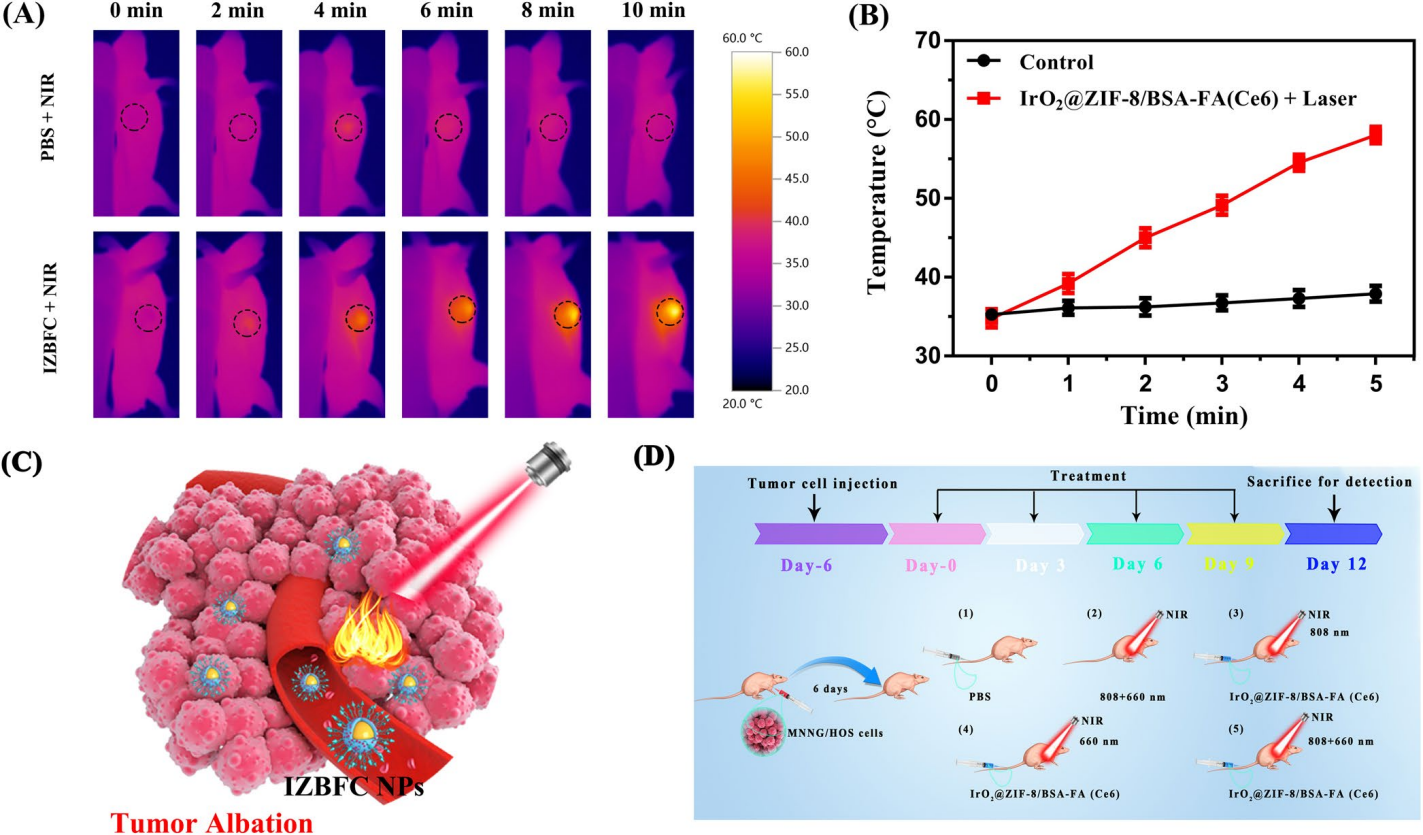
In vivo photothermal effect of the IZBFC NPs. (A) In vivo thermal imaging of MNNG/HOS tumor-bearing mice after intravenous injection of PBS buffer and IZBFC under an 808 nm laser irradiation for 10 min. (B) The time-dependent temperature evaluation profile of mice after treated with PBS and 808 nm laser irradiation. (C) Schematic illustration of in vivo therapy process in tumor-bearing mice. (D) Schematic illustration to show induced PTT/PDT combinatorial effect on tumor ablation.

Inspired by the in vitro results, we next investigated the NIR-triggered photothermal capacity of IZBFC NPs in MNNG/HOS cell bearing Balb/c mice. As depicted in Figure 6(A), the thermal imaging indicated that the mice treated with IZBFC exhibited obvious increases in the temperature of tumor sites, which can achieve about 58.1 °C upon laser irradiation (Figure 6(B)), thus capable of ablating and destructing tumor cells irreversibly. These results demonstrated that the IZBFC NPs could serve as an effect photothermal agent for PTT owing to its excellent photothermal capacity in vivo (Figure 6(C)).

### In vivo synergistic therapeutic efficacy

**Figure 7.**
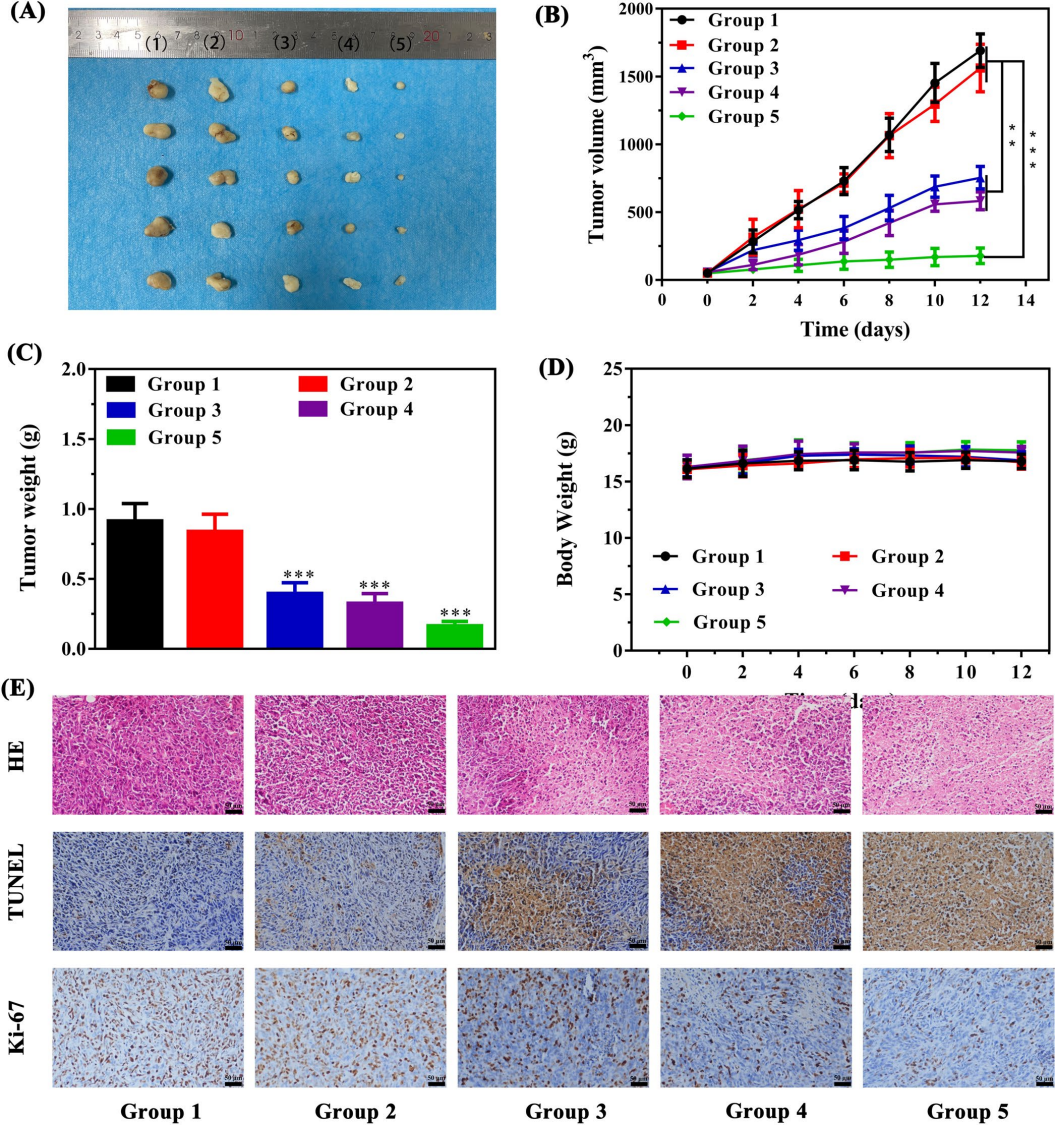
Evaluation of the anti-tumor effects of in vivo photothermal-photodynamic therapy. (A) The representative photograph of excised tumors from treated mice in each group (Groups included (1) control, (2) 808 + 606 nm laser irradiation, (3) IZBFC + 808 nm laser irradiation, (4) IZBFC + 660 nm laser irradiation, and (5) IZBFC + 808 nm + 660 laser irradiation, respectively). (B) Tumor volume growth curves of tumor-bearing mice in each group. (C) Tumor weights were measured after different treatments. (D) The body weight of MNNG/HOS tumor-bearing mice in each group during the treatment periods. (E) H&E, TUNEL and antigen Ki-67 immunohistochemistry of tumor slices in different groups.

Encouraged by the promising results of combination therapy in vitro and the prominent biocompatibility, animal models were established as described above to confirm the IZBFC NPs mediated synergistic PTT and PDT effects. The tumor therapeutic process was depicted in Figure 6(D). As illustrated in Figure 7(A–C), negligible inhibition effect on tumor growth was observed in both PBS and 808 + 660 nm laser irradiation group, which demonstrated that laser exposure alone showed no obvious reductions in tumor growth. The PTT-PDT combination therapy groups exhibited slight tumor growth inhibition effects compared with that of control and laser only group, suggesting that the damage to tumor cells caused by a single dose of PTT and PDT was limited. As expected, obvious inhibitory effect on tumor growth was observed after treating with the combined therapy, indicating a promising combined anti-tumor effect arising from synergistic PTT and PDT. Meanwhile, the body weight of all groups had no obvious changes (Figure 7(D)) during the whole therapeutic period, suggesting that there was no significant toxicity of our as-synthesized NPs.

After the completion of therapeutic course, the mice were sacrificed, and the tumor tissues and major organs were collected for histopathological analysis (Figure 7(E)). H&E staining of tumor sections in PTT-PDT group showed cytoplasm leakages and nucleus shrinkages, suggesting that abundant tumor tissue necrosis. TUNEL staining exhibited an increase in the apoptotic rate of the tumors in the PTT-PDT group. Moreover, Ki-67 staining showed that cell proliferation in the PTT-PDT group was significantly lower, attesting that the tumor growth has been thoroughly inhibited in the PTT-PDT group. Furthermore, in vivo toxicity in major organs in each group were also assessed by H&E staining (Figure S10). Clearly, there was no detectable pathological changes in all groups, further demonstrating the excellent biosafety and biocompatibility in vivo of our versatile nanoplatform. Meanwhile, the blood biochemistry results demonstrated all the measured parameters were in the normal range for the NPs-treated mice group and control group (Figure S11), further proving their favorable biocompatibility and biosafety.

### In vivo blood circulation and biodistribution

Inspired by the prominent in vivo tumor growth inhibition of IZBFC, we proceeded to investigated the blood circulation profiles at different time points. As shown in Figure S12, IZBFC exhibited a blood retention of 8.9% ID/g at 24 h following tail vein injection, and blood circulation half-life time of IZBFC was determined to be 4.36 h. The prolonged half-time of blood circulation also promoted the tumor accumulation of IZBFC, which could be attributed to the EPR effect and FA-mediated active targeting effects, thus inducing the higher uptake of tumor. In addition, the biodistribution of IZBFC NPs after intravenous injection for 12 h and 24 h was further evaluated by ICP-AES test. The biodistribution assay indicated that the IZBFC NPs could effectively accumulate in tumor sites and achieved 9.7% ID/g at 24 h following tail vein injection (Figure S13).

## Conclusion

Taken together, we have successfully designed and developed an intelligent drug delivery system based IZBF NPs which can not only achieve stimulative responsive drug delivery but also amplified PTT-PDT mediated ROS cell-killing capacity. Meanwhile, IZBF NPs capable of catalyzing H_2_O_2_ decomposition to produce O_2_, improving hypoxia microenvironment of tumor and enhancing the therapeutic effects of PDT. The as-synthesized IZBF NPs showed excellent biocompatibility, prominent photostability, and efficient photothermal conversion efficiency under laser irradiation. We have also utilized this NPs as an effective nanocarrier to loaded with Ce6, which provided a high capacity for drug loading, and the drug release could be efficiently triggered by acidic pH and NIR laser irradiation. More importantly, the synergistic therapeutic effects of IZBFC NPs coupled with NIR laser irradiation has been validated both in vitro and in vivo, which significantly showed better antitumor performance than that of the monotherapy groups. Besides, in vivo experiments demonstrated that efficient accumulation of NPs in tumors, likely owing to EPR effect together with FA-mediated targeting effect facilitating the enrichment of NPs into tumor site. Overall, the present work presented new strategies for the construction of an intelligent nanoplatform integrating PTT, PDT and tumor oxygenation into a single nanoplatform, which exhibited enormous potential for cancer therapy.

## Supplementary Material

Supplemental Material
