# Supplementary material for: Synthesis of dual-stimuli responsive metal organic framework-coated iridium oxide nanocomposite functionalized with tumor targeting albumin-folate for synergistic photodynamic/photothermal cancer therapy
Source: Drug Deliv. 2022 Sep 26;29(1):3142–54. doi: 10.1080/10717544.2022.2127973 (PMC9542428; doi:10.1080/10717544.2022.2127973)
Supplement: Supplemental Material [file IDRD_A_2127973_SM8806.docx]

**Supporting Information**

**Synthesis of dual-stimuli responsive metal organic framework-coated iridium oxide nanocomposite functionalized with tumor targeting albumin-folate for synergistic photodynamic/photothermal cancer therapy**

Xiangtian Deng^1,2^, Renliang Zhao^1,2^, Qingcheng Song^5^, Yiran Zhang^4^, Haiyue Zhao^4^, Hongzhi Hu^6^, Zhen Zhang^1,2^, Weijian Liu^6, #^, Wei Lin^3, #^, Guanglin Wang^1, 2, #^

^1^Trauma medical center, Department of Orthopedics surgery, West China Hospital, Sichuan University, Chengdu 610041, China.

^2^Orthopedics Research Institute, Department of Orthopedics, West China Hospital, Sichuan University

^3^Department of Gynecology, West China Second Hospital, Sichuan University, Chengdu, China

^4^School of Medicine, Nankai University, Tianjin, 300071, China

^5^Department of Orthopaedic Surgery, The Third Hospital of Hebei Medical University, Shijazhuang, 050051, China.

^6^Department of Orthopaedics, Union Hospital, Tongji Medical College, Huazhong University of Science and Technology, Wuhan 430022, China.

**Disclosure statement**

There are no conflicts of interest to declare.

**Funding**

None.

**Author contribution**

Xiangtian Deng and Renliang Zhao equally contribution

**Corresponding author**

^#^ Correspondence to Weijian Liu, Wei Lin, and Guanglin Wang.

1. **Materials**

Iridium chloride (IrCl_3_), polyvinylpyrrolidone (PVP), sodium hydroxide (NaOH), zinc nitrate hexahydrate (Zn(NO_3_)_2_·6H_2_O, 99.99%) and 2-methylimidazole (2-MIM, 98%) were commercially obtained from Aladdin Bio-Chem Technology Co., Ltd. (Shanghai, China). Bovine serum albumin (BSA, 99%) and Folic acid (FA, 98%) were purchased from Beijing Solarbio Technology Co., Ltd. All other chemicals and reagents were commercially available and used as received without further purification.

1. **Characterization**

The transmission electron microscopy (TEM, FEI Talos F200X, 200 kV) was used for characterizing the morphologies of NPs. Scanning electron microscopy (SEM) images was performed by a transmission electron microscope (ZEISS Gemini 300). Hydrodynamic diameters and zeta potential were evaluated by dynamic light scattering (DLS) using a Zetasizer Nano ZS90 (Malvern, UK). The UV-vis absorption spectra were detected by a UV-vis spectrometer (UV-3600, Shimadzu, Japan). Chemical composition was characterized by X-ray photoelectron spectroscopy (XPS, Thermo Scientific K-Alpha). The chemical information of the IrO2@ZIF-8/BSA-FA were examined by Fourier transform infrared (FTIR, Perkin Elmer Spectrum 100) spectrometer, respectively.

1. **Supplementary figures**


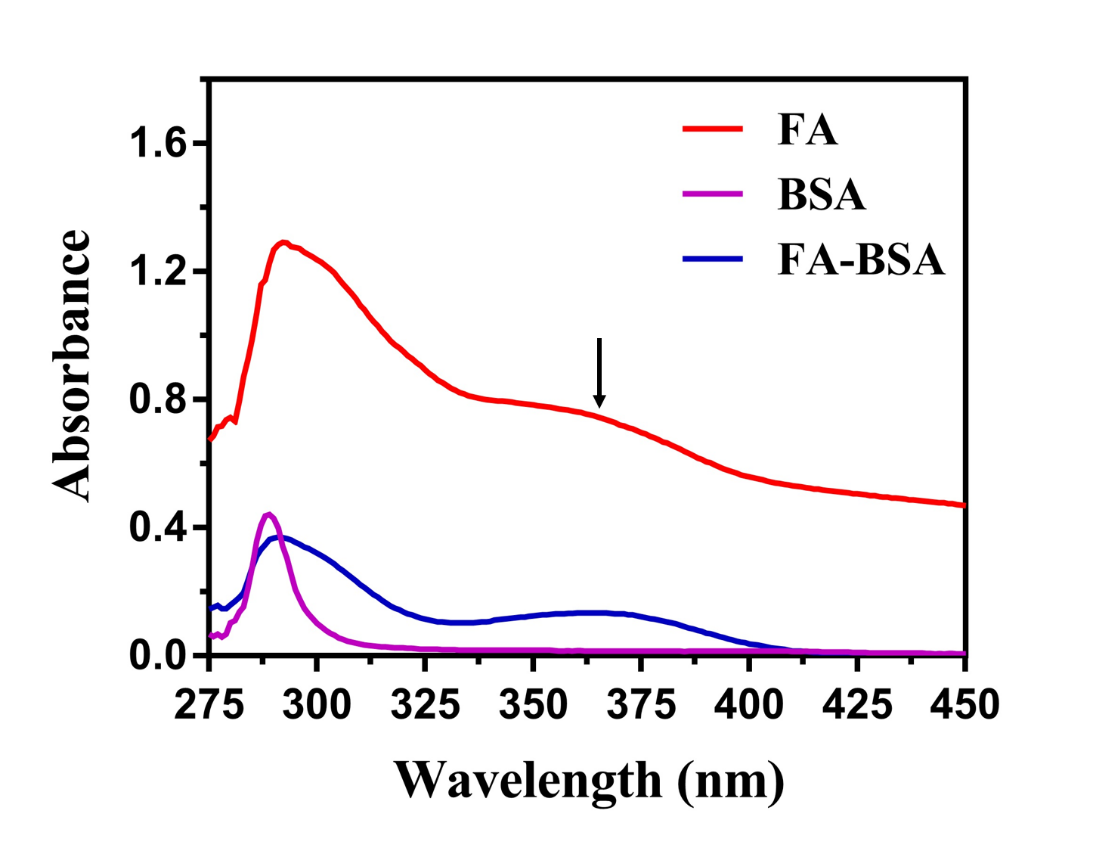
+

**Figure S1** UV-Vis absorbance spectra of FA, BSA, and FA-BSA. The obvious absorbance at 363 nm of FA-BSA spectra confirmed the successful synthesis of FA-BSA.


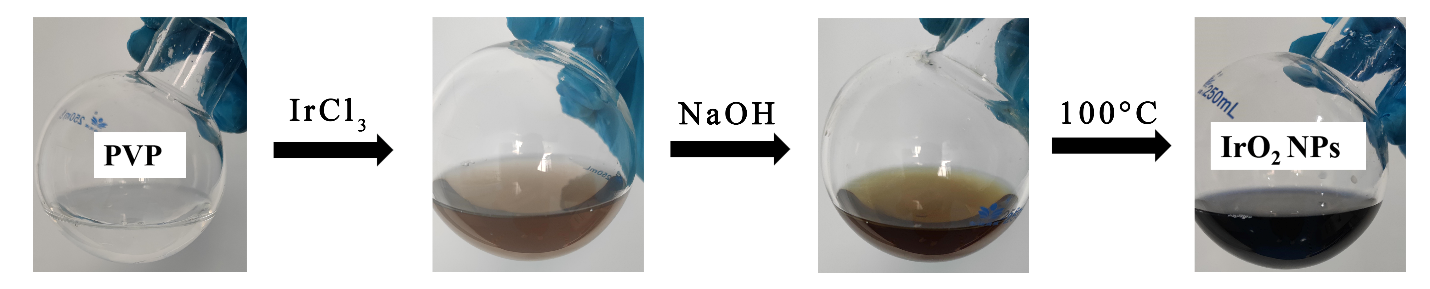


**Figure S2** The synthetic process of IrO_2_ NPs.


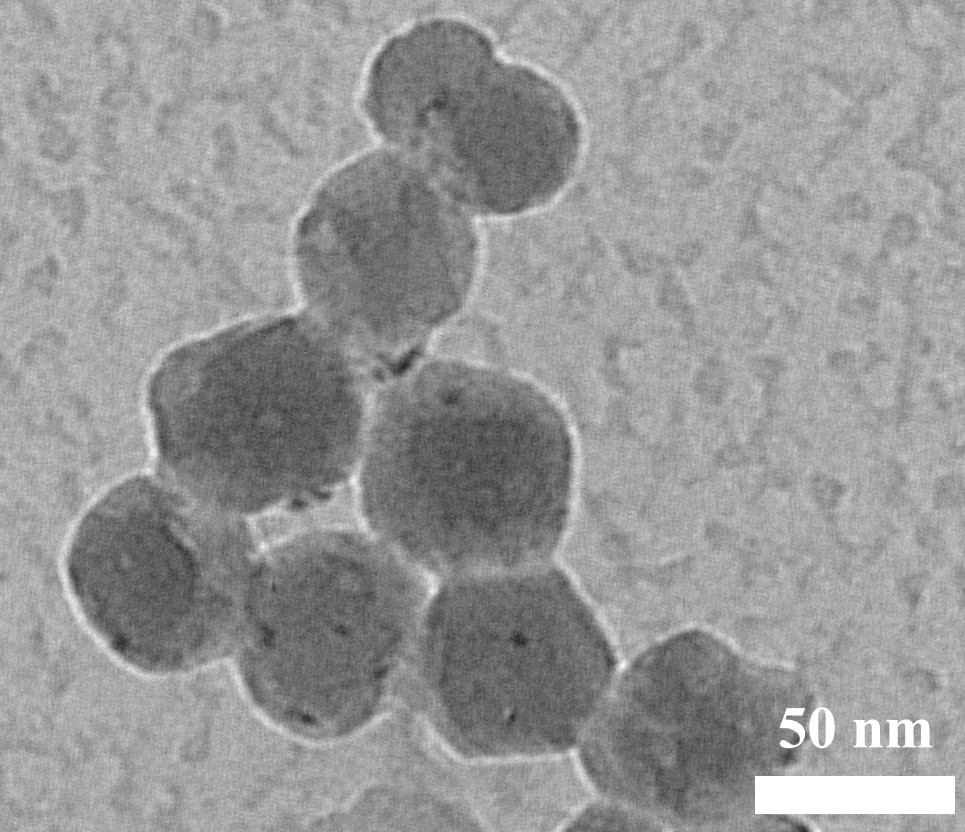


**Figure S3** TEM images of IrO_2_@ZIF-8 NPs.


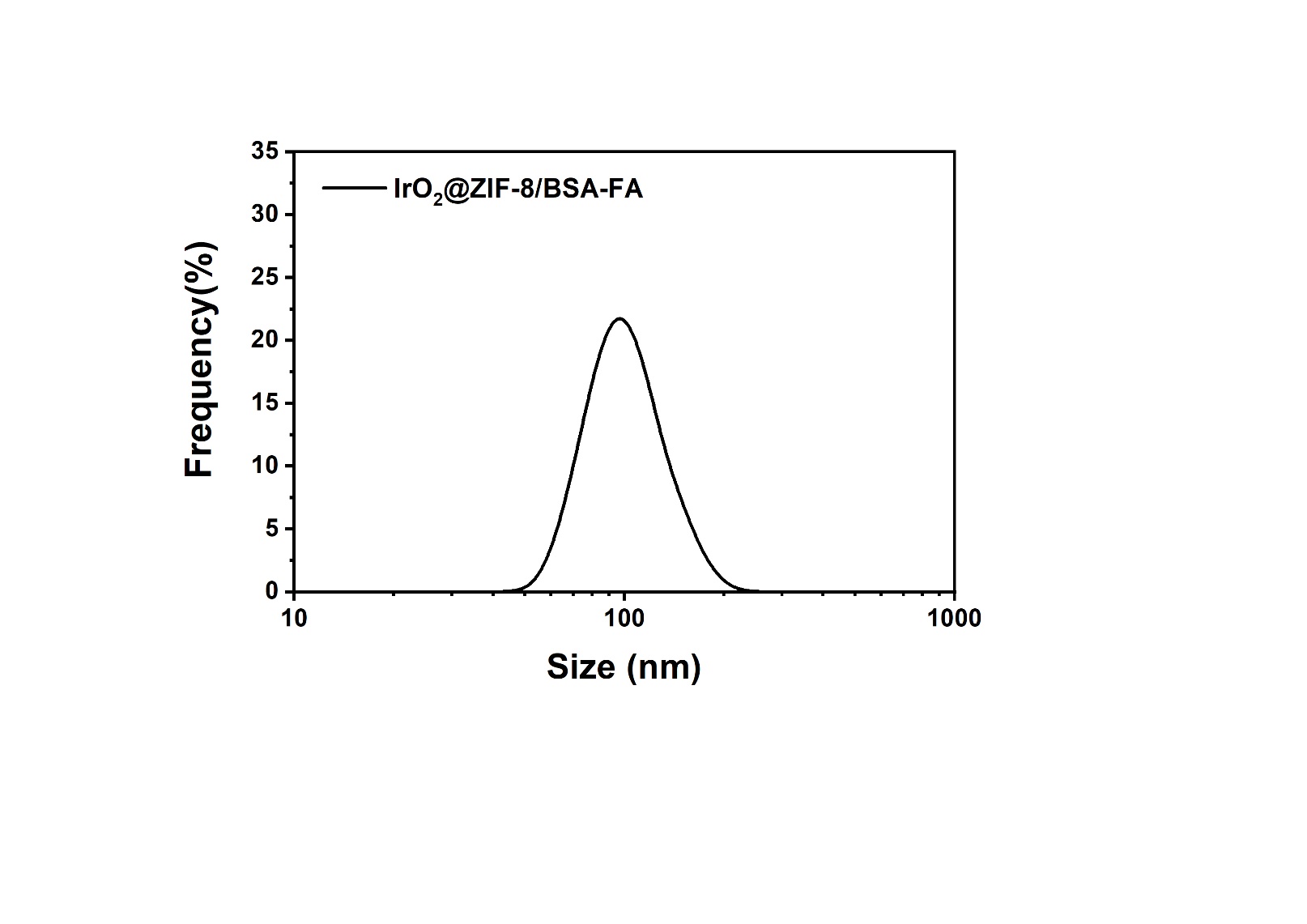


**Figure S4** Hydrodynamic diameter of IrO_2_@ZIF-8/BSA-FA NPs.


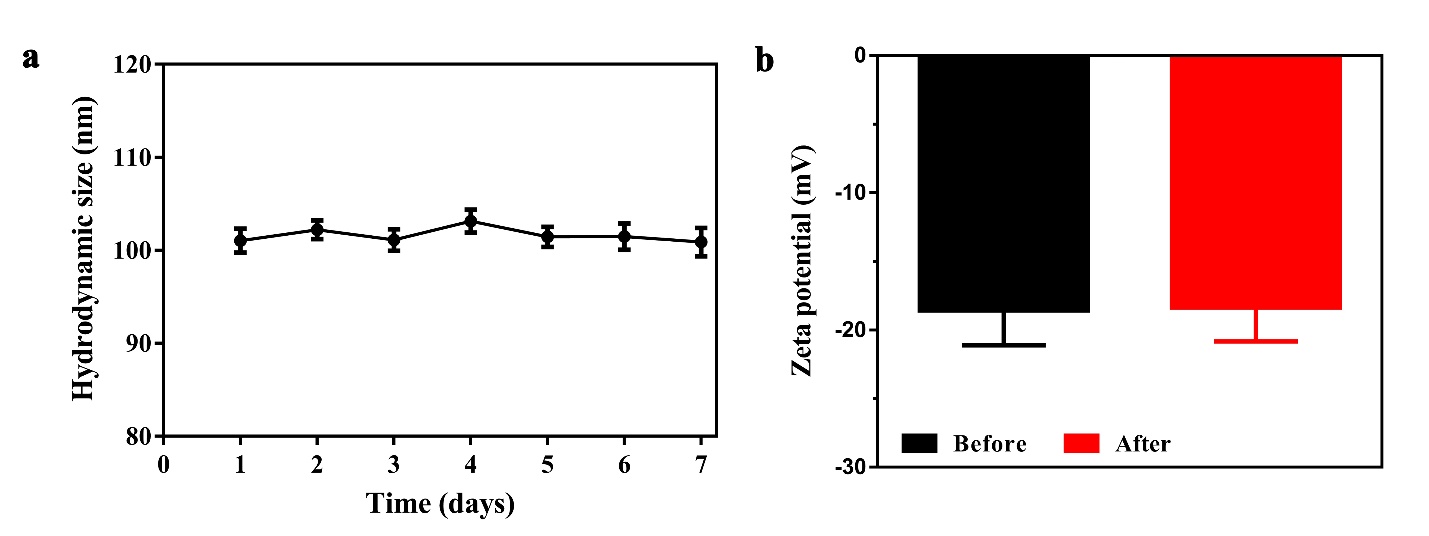


**Figure S5** (a) Hydrodynamic diameter of IZBF within 7-day dialysis in PBS buffer (pH 7.4). (b) Zeta potentials of aqueous IZBF dispersion before and after 7 day’s dialysis in PBS buffer (pH 7.4). Data shown as mean ± SD, n = 3 per treatment.


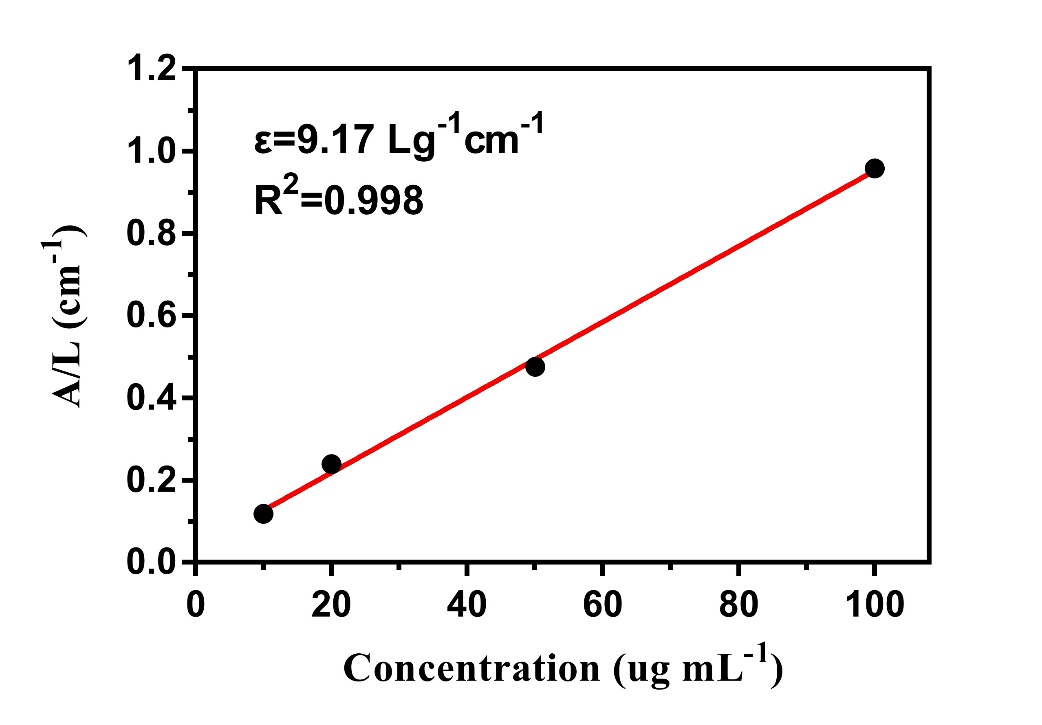


**Figure S6** Extinction coefficient of the NPs aqueous solution at 808 nm. Normalized absorbance intensity at 808 nm divided by the characteristic length of the cell (A/L) at varied concentrations.


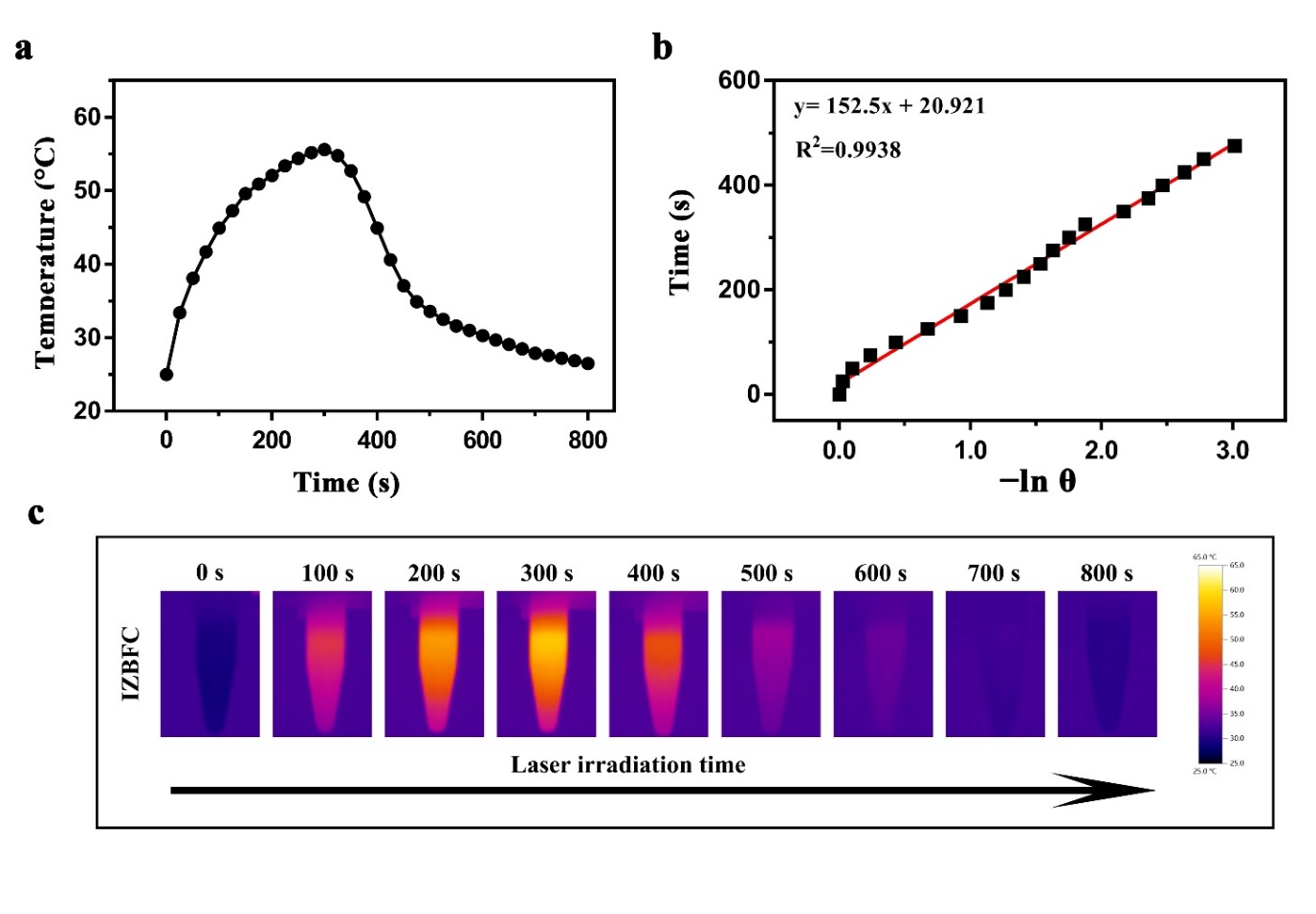


**Figure S7** Photothermal-conversion performance of IrO_2_@ZIF-8/BSA-FA NPs. (a) Temperature change of a IrO_2_@ZIF-8/BSA-FA dispersion (3.0 mM with respect to Ir) exposed to an 808 nm laser irradiation. (b) The time constant for heat transfer of the IrO_2_@ZIF-8/BSA-FA dispersion, calculated by plotting time versus ln θ during the cooling process. (c) The photothermal images during the heating and cooling process.


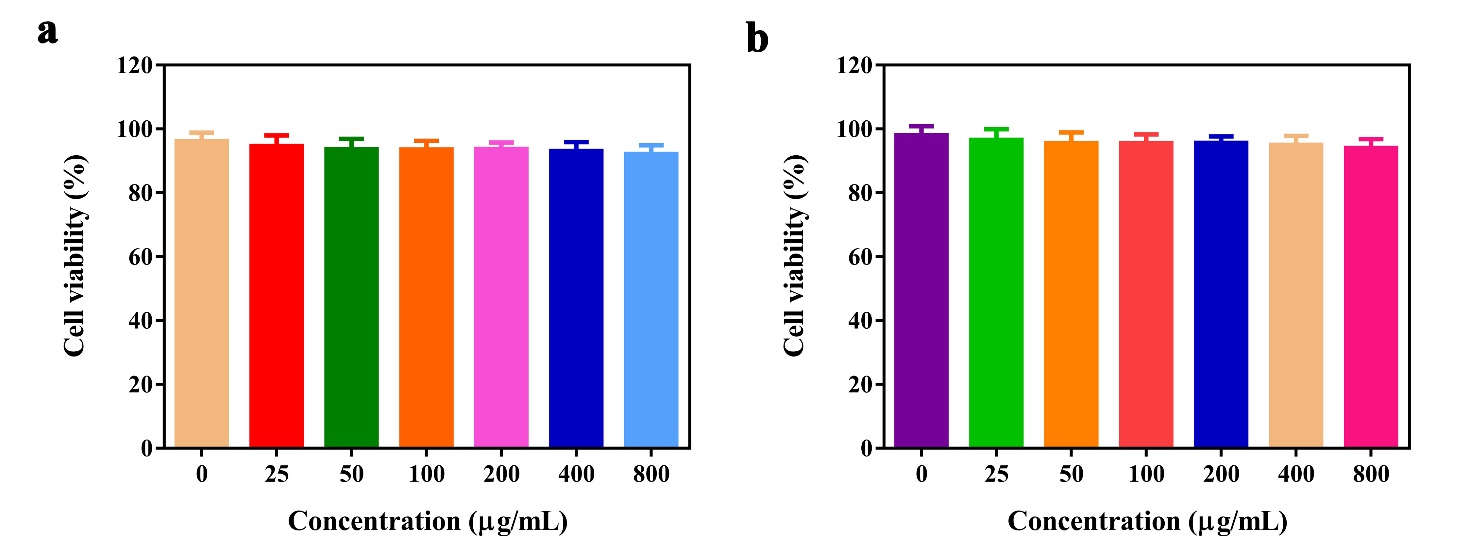


**Figure S8 (a)** Cell viability of BMSCs after 24 h of treatments with different concentrations of IrO_2_@ZIF-8 NPs. **(b)** Cell viability of BMSCs after 24 h of treatments with different concentrations of IrO_2_@ZIF-8/BSA-FA NPs.


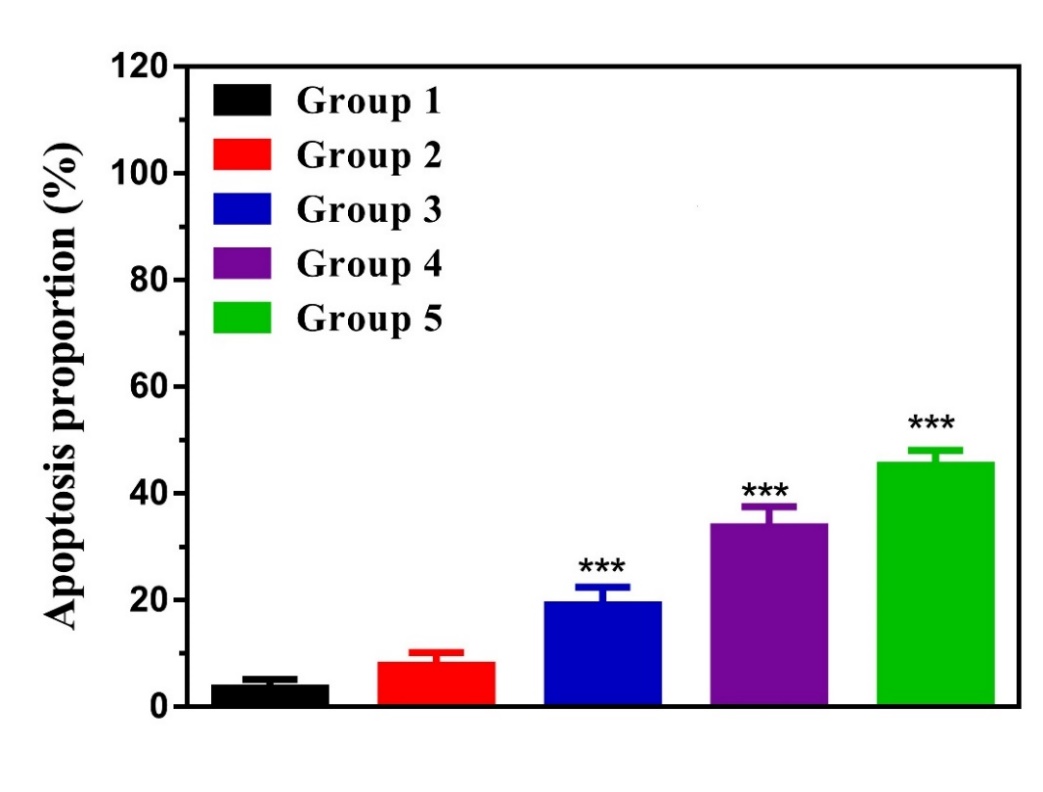


**Figure S9** Apoptosis ratios of cells after corresponding treatment was quantified.


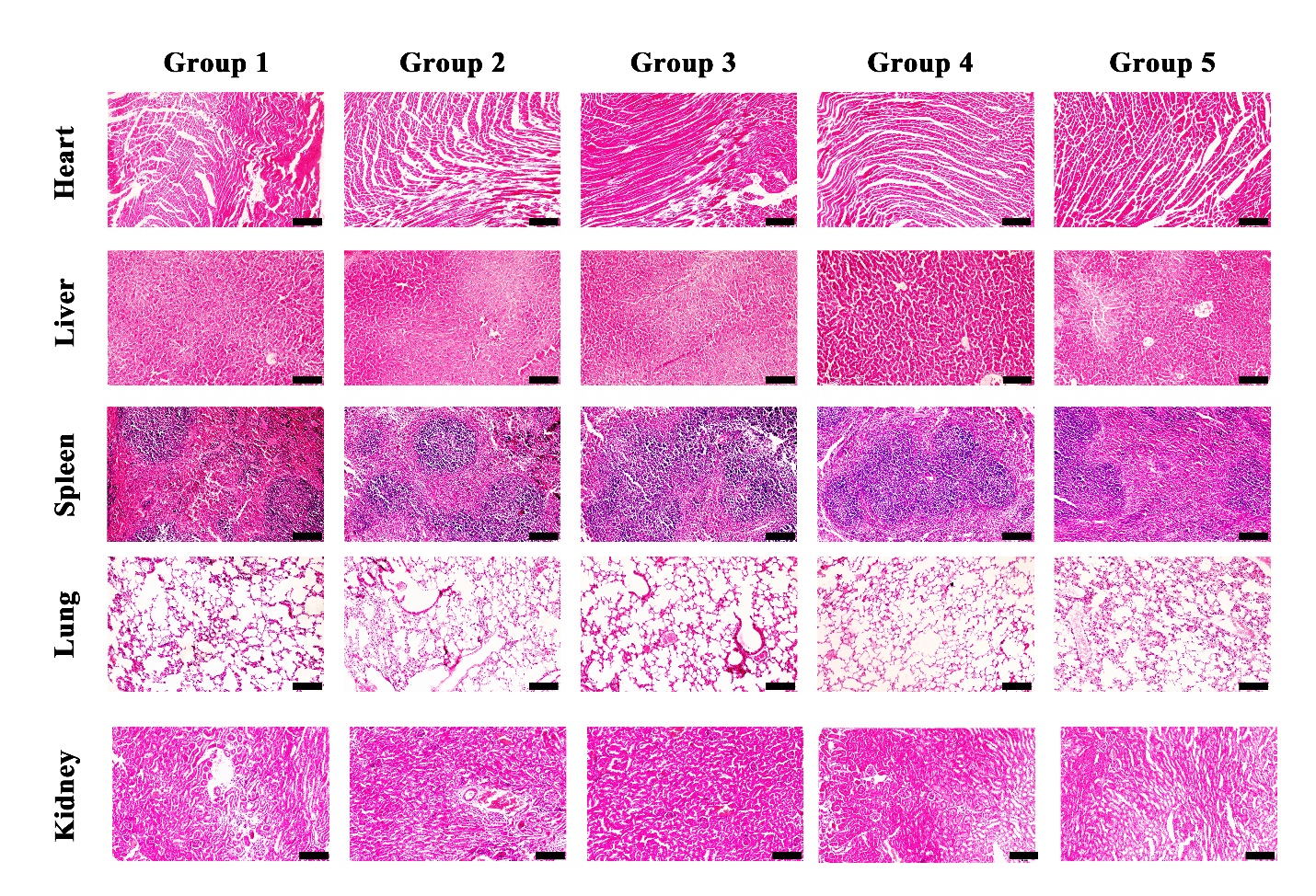


**Figure S10** H&E staining images of major organs (heart, liver, spleen, lung, and kidney) collected from mice treated with (1) PBS, (2) 808 + 660 nm laser irradiation, (3) IrO_2_@ZIF-8/BSA-FA (Ce6) + 808 nm, (4) IrO_2_@ZIF-8/BSA-FA (Ce6) + 660 nm, and (5) IrO_2_@ZIF-8/BSA-FA (Ce6) + 808 nm + 660 nm lasers, respectively. Scale bars = 100 μm.


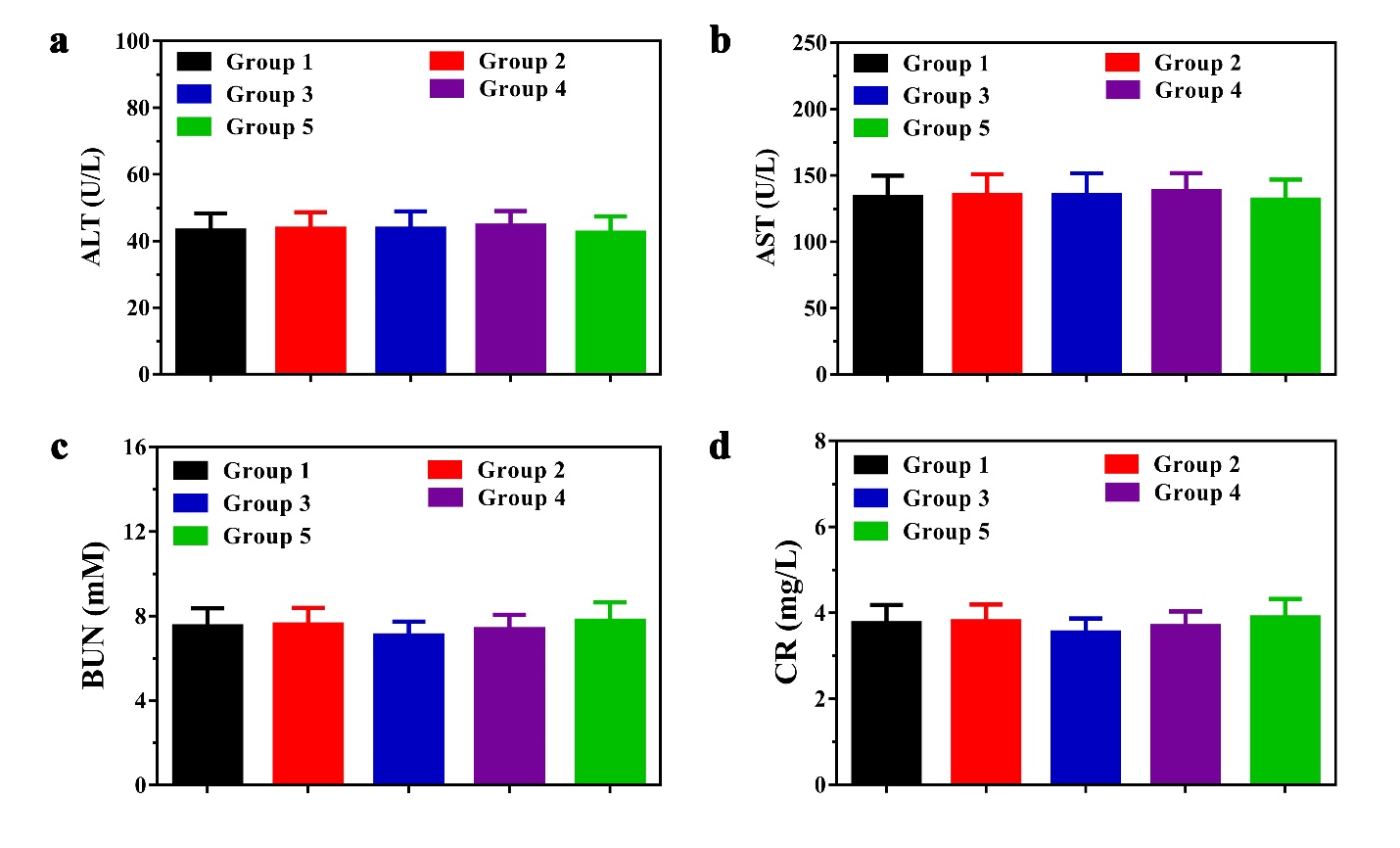


**Figure S11** Blood biochemistry evaluation of Balb/c mice treated with IrO_2_@ZIF-8/BSA-FA (Ce6) NPs. **a, b** Serum levels of ALT and AST (liver function index). **c, d** Serum levels of BUN and CR (kidney function index).


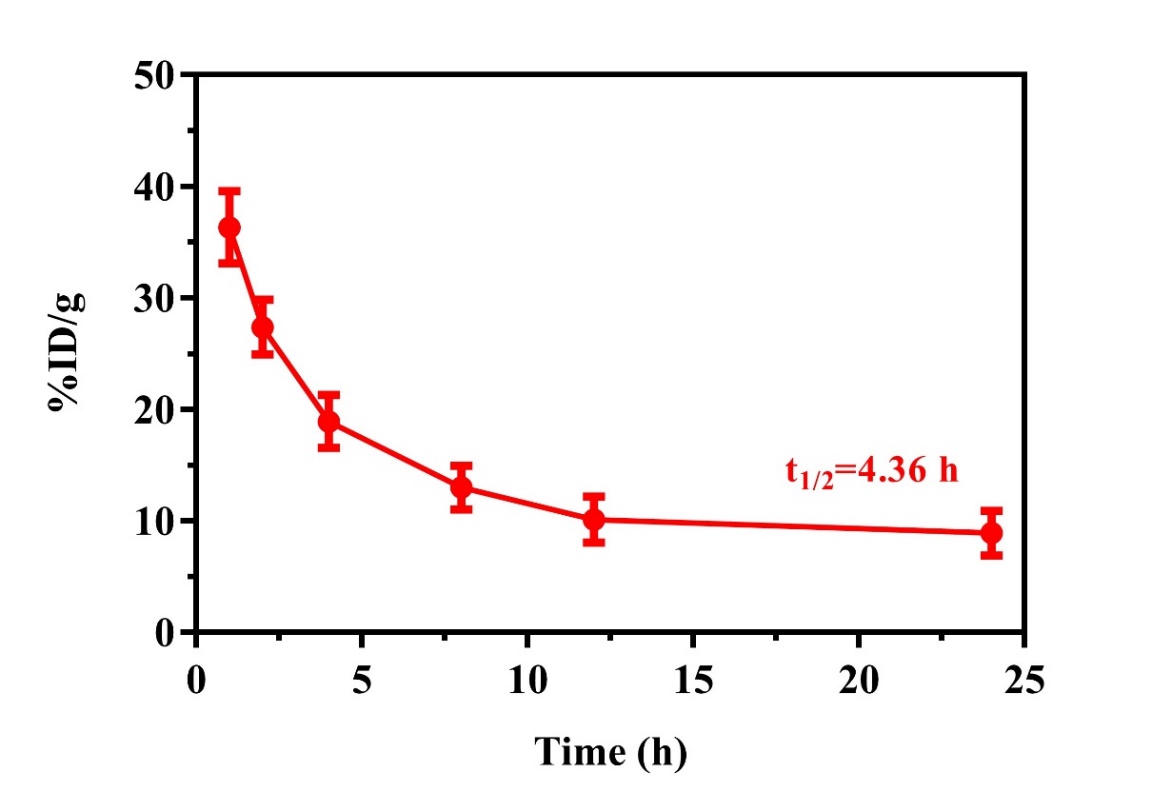


**Figure S12** Blood circulation half-time of IZBFC over a span of 24 h after intravenous injection into MNNG/HOS tumor-bearing mice.


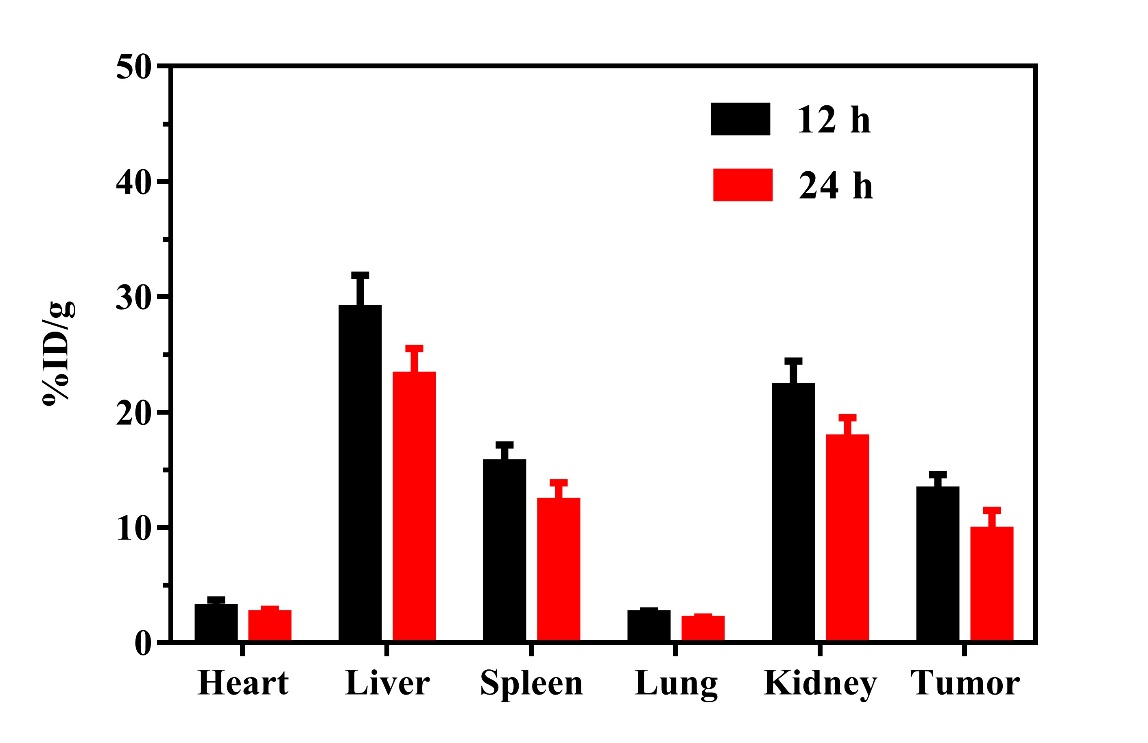


**Figure S13** Biodistribution of major organs and tumor tissue at 12 h and 24 h post-injection
